# MiR-122-5p regulates the mevalonate pathway by targeting p53 in non-small cell lung cancer

**DOI:** 10.1038/s41419-023-05761-9

**Published:** 2023-04-01

**Authors:** Yu-kun Zheng, Zhong-shi Zhou, Guang-zhong Wang, Ji-yuan Tu, Huan-bo Cheng, Shang-zhi Ma, Chang Ke, Yan Wang, Qi-pan Jian, Yu-hang Shu, Xiao-wei Wu

**Affiliations:** 1 Department of Thoracic Surgery, Tongji Hospital, Tongji Medical College, Huazhong University of Science and Technology, Wuhan, 430030 China; 2 College of Pharmacy, Hubei University of Chinese Medicine, Wuhan, 430065 China; 3 Hubei Engineering Technology Research Center of Chinese Material Medical Processing Technology, Wuhan, 430065 China

**Keywords:** Oncogenes, Non-small-cell lung cancer

## Abstract

The 5-year survival rate of non-small cell lung cancer (NSCLC) patients is very low. MicroRNAs (miRNAs) are involved in the occurrence of NSCLC. miR-122-5p interacts with wild-type p53 (wtp53), and wtp53 affects tumor growth by inhibiting the mevalonate (MVA) pathway. Therefore, this study aimed to evaluate the role of these factors in NSCLC. The role of miR-122-5p and p53 was established in samples from NSCLC patients, and human NSCLC cells A549 using the miR-122-5p inhibitor, miR-122-5p mimic, and si-p53. Our results showed that inhibiting miR-122-5p expression led to the activation of p53. This inhibited the progression of the MVA pathway in the NSCLC cells A549, hindered cell proliferation and migration, and promoted apoptosis. miR-122-5p was negatively correlated with p53 expression in p53 wild-type NSCLC patients. The expression of key genes in the MVA pathway in tumors of p53 wild-type NSCLC patients was not always higher than the corresponding normal tissues. The malignancy of NSCLC was positively correlated with the high expression of the key genes in the MVA pathway. Therefore, miR-122-5p regulated NSCLC by targeting p53, providing potential molecular targets for developing targeted drugs.

## Introduction

Lung cancer is the most common malignancy of the respiratory system and the leading cause of cancer-related death [1]. Non-small cell lung cancer (NSCLC) accounts for approximately 85% of all lung cancer cases [2], with a 5-year survival rate of patients is less than 15% [3]. Some studies showed that microRNAs (miRNAs) are involved in the development of NSCLC [4, 5]. Indeed, miRNAs are the key endogenous factor that induces cancer regulating the expression of the corresponding target molecules and participating in a series of biological processes such as cell proliferation, migration, and apoptosis [6]. Our research group has been engaged in basic research associated with NSCLC. Our previous study found that miR-122-5p is involved in the progression of NSCLC. Moreover, an interaction between miR-122-5p and the cancer protein p53 was determined by bioinformatics analysis and luciferase reporter experiments [7]. However, the mechanism used by miR-122-5p to regulate the effect of p53 on the occurrence and development of NSCLC remains unclear.

p53 is one of the main cancer suppressors involved in the regulation of cell cycle arrest, apoptosis, and genome stability [8]. It is closely related to NSCLC, is one of the most frequently mutated genes in this cancer type, and is used as a biomarker to predict the prognosis of NSCLC patients [9, 10]. The p53 knockdown promotes the proliferation and migration of NSCLC cells [11]. p53 is also involved in the lipid metabolism of cancer cells. Studies showed that p53 targets the ATP-binding cassette transporter A1 (ABCA1) to inhibit the maturation of sterol regulatory element-binding protein 2 (SREBP2) [12]. However, its role in the lipid metabolism of NSCLC has not been reported.

SREBP2 is a master regulator of the mevalonate (MVA) pathway [13, 14], which in turn is an important biological pathway that allows cells to produce sterols (such as cholesterol) and non-steroidal isoprenoids [15]. The expression of its key genes, such as HMGCS1, HMGCR, and FDFT1, is associated with the development of tumors. HMGCS1 promotes lung tumor metastasis in vivo, and the reduction of its expression inhibits cancer cells growth [16, 17]. HMGCR is the most important rate-limiting gene in the MVA pathway. Therefore, targeting HMGCR is an effective therapeutic strategy for cancer treatment [16, 18]. FDFT1 plays an important role in proliferation, invasion, and metabolic reprogramming, and the decrease of its expression reduces cholesterol production [19, 20]. However, the role of the MVA pathway in NSCLC remains unclear.

This study investigated the effect of miR-122-5p on the MVA pathway in NSCLC cells with molecular biology experiments. The results revealed that miR-122-5p is crucial in maintaining tumor cell proliferation. The importance of the MVA pathway in the occurrence and development of NSCLC revealed that it led to more severe NSCLC progression. Thus, it provided a theoretical basis for using miR-122-5p as a cancer marker and targeting the MVA pathway in the treatment of NSCLC. The current research has crucial scientific significance, a clinical value for developing targeted therapeutic interventions and effective treatments.

## Material and methods

### Human samples

Tumor samples from NSCLC patients and corresponding normal tissue samples (*n* = 18) were provided by the Tongji Affiliated Hospital of Huazhong University of Science and Technology. This study was reviewed and approved by the Medical Ethics Committee of Tongji Hospital, Affiliated with Tongji Medical College, Huazhong University of Science and Technology (medical ethics committee approval number: TJ-IRB20220639), and informed consent was obtained from all subjects.

### Cell lines and culture conditions

Human NSCLC cells A549 and human normal lung epithelial cells BEAS-2B were provided by the Tongji Hospital, Affiliated with Tongji Medical College of Huazhong University of science and technology. We purchased human NSCLC cells H1944 (cat#CL-0632) from Procell. A Mycoplasma contamination test was carried out, which was negative. A549 cells were routinely cultured in F12 medium (Procell, cat#PM150312), BEAS-2B cells were routinely cultured in DMEM with high glucose (Gibco, cat#11965092), and the H1944 cells were routinely cultured in RPMI-1640 medium (Procell, cat#PM150110). All the media were supplemented with 1% penicillin and streptomycin solution (Beyotime, cat#ST488) and 10% fetal bovine serum (Gibco, cat#A3160902). The cells were incubated at 37 °C under 5% CO_2_. Nutlin-3 (Glpbio, cat#GC16051) and Simvastatin (SIM, Selleck, cat#S1792) powder were dissolved in DMSO to prepare a stock solution. The working solution was diluted to 10 μM in the medium. DL-Mevalonolactone (DL-MVA) (Molnova, cat#M15570) and Methoxyestradiol (2-MeOE2, Selleck, cat#S1233) solutions were dissolved in a medium and respectively diluted to 100 μM and 10 μM.

### miRNA inhibitor, miRNA mimic, si-RNAs, and cell transfection

A549 cells were seeded in 6-well or 96-well plates at a density of 5 × 10^3^ cells/well, or 1.5 × 10^5^ cells/well, respectively and transfected with a serum-free medium containing RNA oligomer-LipoFectMAX^TM^ (GeneCodex, cat#T003) complexes. The medium was replaced using a complete medium after 6 h. The transfection efficiency peaked at 72 h, and subsequent experiments were carried out. Details of the primer sequences of miR-122-5p inhibitor, miR-122-5p mimic, si-p53, and p53 Target sequence are provided in the Supplementary Information (Supplementary Table. S2).

### Cell Counting Kit 8 (CCK-8) assay

NSCLC cells were seeded into a 96-well plate at 5 × 10^3^ cells/well density and were treated using the corresponding reagents. This included, 122-5p inh. combined with si-p53 or 122-5p inh. alone or 122-5p mim. combined with Nutlin-3 or 122-5p mim. alone after the cells adhered to the bottom of the wells. Ten microliters of CCK-8 reagent were added to each well after 72 h, and the cells were incubated for 30 min. For normal lung cells BEAS-2B, 122-5p mim. and 122-5p inh. were given other than 1,2,4,8 times the dose indicated by the reagent. Moreover, the other treatment methods were the same as NSCLC cells. The absorbance (OD) was measured at 450 nm with a microplate reader (Biotek, USA) based on the manufacturer’s instructions for the CCK-8 kit (Beyotime, cat#C0038).

### Colony formation assay

The treated NSCLC cells, such as 122-5p inh. combined with si-p53 or 122-5p inh. alone or 122-5p mim. combined with Nutlin-3 or 122-5p mim. alone were seeded in 6-well plates at 1 × 10^3^ cells/well [21] density. After two weeks, the cells were fixed with paraformaldehyde at room temperature, washed and stained with 0.05% crystal violet, rewashed, and dried. Images were taken using a white light projector, using the number of colonies formed under each condition was counted under a microscope (Olympus, Japan).

### Cell scratch assay

NSCLC cells were seeded onto 6-well plates at a certain density and treated with 122-5p inh. combined with si-p53 or 122-5p inh. alone or 122-5p mim. combined with Nutlin-3 or 122-5p mim. alone. When the cell production fusion rate achieved 90% or more, a sterile pipette tip helped scratch an even straight line in each well. Then, the cells were washed twice with PBS to remove the scratched cells. Then, serum-free medium was added, and they were incubated and observed every 12 h up to 24 h. Images were taken at 40× magnification with the microscope at the same position. The area between the two borders of the scratch was measured using Image J (NIH image software), and the inhibition rate of cell migration was determined. The calculation formula is as follows: cell migration inhibition rate = (the area between the two borders of the scratch in the experimental group - the area between the two borders of the scratch at each time point in the experimental group) / (the area between the two borders of the initial scratch in the control group - the area between the two borders of the scratch at each time point in the control group) × 100%.

### Apoptosis assays

NSCLC cells were lysed using trypsin without EDTA, harvested, and processed according to the instructions of the Apoptosis Kit (Biosciences, cat#AB1926A1). The cells were washed twice with PBS pre-cooled at 4 °C, and the concentration was adjusted to 1 × 10^6^ cells/mL with 1 × Annexin-binding buffer. A total of 100 μL of fine run suspension was placed into a 1.5 mL centrifuge tube, 5 μL FITC Annexin V and 1.5 μL Pl were added, and the mixture was left at room temperature for 15 min in the dark. A total of 400 μL 1 × Annexin-binding buffer was added, and then the mixture was gently mixed and placed on ice. Cell apoptosis was detected within 1 h using a flow cytometer (BD Biosciences, USA). The data were analyzed using Flow Jo V10 software.

### RT-qPCR

A549 cells were treated using 122-5p inh. combined with si-p53 or 122-5p inh. combined with DL-MVA or 122-5p inh. alone or 122-5p mim. alone or Nutlin-3 combined with DL-MVA or Nultin-3 alone or si-p53 alone or 2-MeOE2 combined with 122-5p mim. or 2-MeOE2 alone. Moreover, total RNA was extracted from the above A549 control and A549 with BEAS-2B untreated groups using Trizol based on the manufacturer’s instructions (Thermo Fisher Scientific, cat#10296010). The nine genes of the MVA pathway were designed using the Nucleotide function of the NCBI webpage (https://www.ncbi.nlm.nih.gov) to detect the mRNA expression. The primer sequence table is abbreviated in Supplementary Table 3. Total RNA was reverse transcribed into cDNA using a reverse transcription Kit (Vazyme, cat#R223-01), and the amplification system was configured with an amplification kit (Abclonal, cat#RK20429). The primers of miR-122-5p were designed and purchased from the Genecopoeia company to detect miRNA expression. All-in-one™ miRNA qRT-PCR detection kit 2.0 (Genecopoeia, cat#QP115) configuration reversal and amplification system were used. A PCR instrument (Analytik Jena, Germany) was used to amplify. We performed a three-step PCR reaction for mRNA expression: (1) Pre-denaturation at 95 °C for 1 min. (2) Denaturation at 95 °C for 20 s and annealing/extension at 60 °C for 45 s. (3) Reaction at 95 °C for 1 min, and the melting curve was calculated after 40 cycles. A two-step PCR reaction was performed for miRNA expression: (1) Pre-denaturation at 95 °C for 10 min. (2) Denaturation at 95 °C for 10 s, annealing at 75 °C for 20 s, extension at 72 °C for 10 s, and the melting curve was determined after 40 cycles. The specificity of the reaction was verified using melting curve analysis. The expression of each gene was determined through the 2^−ΔΔCt^ method depending on the cycle number.

### Western blotting

Western blotting was performed according to the standard protocol. The extracted A549 proteins were loaded onto a 10% gel and separated using sodium dodecyl sulfate-polyacrylamide gel electrophoresis (SDS-PAGE). Then, they were transferred to a polyvinylidene fluoride (PVDF) membrane with pores of 0.45 μM diameter. The membrane was treated using 5% skimmed milk for 1 h and incubated with the following primary antibodies at 4 °C: anti-β-Actin (Cell Signaling, cat#12620, dilution 1/10,000 in 5% milk/TBS-T) was used as the loading control, anti-p53 (Proteintech, cat#10442-1-AP, dilution 1/2000 in 5% milk/TBS-T), anti-ABCA1 (Abcam, cat#ab66217, dilution 1/1000 in 5% milk/TBS-T), anti-SREBP2 (Proteintech, cat#28212-1-AP, dilution 1/1000 in 5% milk/TBS-T), anti-HMGCS1 (Proteintech, cat#17643-1-AP, dilution 1/1000 in 5% milk/TBS-T), anti-HMGCR (Zenbio, cat#384588, dilution 1/1000 in 5% milk/TBS-T), anti-FDFT1 (Proteintech, cat#13128-1-AP, dilution 1/1000 in 5% milk/TBS-T). Antibodies were washed overnight using TBS-Tween 20 buffer. Then the membrane was treated using the secondary antibody goat anti-mouse IgG antibody (Solarbio, cat#SE131, dilution 1/5000 in 5% milk/TBS-T) or goat anti-rabbit IgG antibody (Solarbio, cat#SE134, dilution 1/5000 in 5% milk/TBS-T) and incubated at room temperature for 1 h. ECL chemiluminescence solution (Solarbio, cat#A6856) was used for exposure and development of the gel imaging system (ProteinSimple, USA). ImageJ software was used to quantify the gray protein bands.

### IHC and HE staining

Sections of paraffin-embedded tumor tissue were deparaffinized and subjected to graded ethanol. Then, they were incubated with antibodies against p53, HMGCS1, HMGCR, and FDFT1 proteins, the same as those used in western blotting. Based on the manufacturer’s instructions, color development was performed using DAB chromogenic kit (Dako, cat#K5007). Hematoxylin (Solarbio, cat#H8070-5g) was used to counterstain for 3 min, followed by a rinse using distilled water, and returned to blue using a blue-returning solution. The coverslip was placed after an additional rinsing. ImageJ software was utilized to detect the AOD of positive areas.

Hematoxylin-eosin (HE) staining was performed using the correspondent kit (Solarbio, cat#G1120). Treated patient tumor and normal tissue sections were incubated in sufficient hematoxylin solution for 5 min, rinsed twice using distilled water to remove the excess staining, and returned to blue using a blue-returning solution. The sections were rewashed with distilled water and dehydrated, then stained with eosin solution for 5 min. The sections were rinsed three times with absolute ethanol, placed in neutral gum, and the morphology was observed under an optical microscope.

### A-CoA and TC detection

A-CoA was measured on A549 cells treated with 122-5p inh. combined with si-p53 or 122-5p inh. alone or Nutlin-3 combined with DL-MVA or Nultin-3 alone and the above A549 corresponding control group with an ELISA detection kit (Elabscience, cat#E-EL-0125C) based on the manufacturer’s instructions. A standard solution or a test sample was added to the microtiter plate, and the sample was incubated at 37 °C for 90 min. The liquid in the well was discarded, and a 100 μL biotinylated antibody/antigen working solution was added. The sample was incubated at 37 °C for 60 min, and washed three times. A total of 100 μL enzyme conjugate working solution was added, and the samples were incubated at 37 °C for 30 min and washed five times. A total of 90 μL of the substrate solution was added, the sample was incubated at 37 °C for 15 min, and 50 μL of stop solution was added. The OD value was measured at 450 nm using a microplate reader. A standard curve calculated the content of A-CoA and compared the differences among the ctrl group, where A549 cells were treated using a medium solution containing 122-5p inh. NC and si-p53 NC or DMSO.

Based on the manufacturer’s instructions, TC was measured on the same outgoing processing group as Acetyl-CoA, with a biochemical kit (Elabscience, cat#E-BC-K109-M). A total of 2.5 μL double-distilled water, standard solution, or test sample was added to the microtiter plate. An amount of 250 μL reaction reagent was added to each well, the sample was incubated at 37 °C for 10 min, and the OD value was measured at 510 nm on the microplate reader. The TC content was determined based on the formula provided by the kit instructions, and the difference between the dosing and control group was compared.

### LC–MS

#### Sample preparation [22]

All the cell samples were transferred into a 2 mL centrifuge tube, and 100 mg glass beads were added. An amount of 1000 µL acetonitrile (ACN): methanol: H_2_O mixed solution (2:2:1, V/V/V) (stored at 4 °C) was accurately added, and the mixture was vortexed for 30 s. The centrifuge tube containing the sample was placed into the 2 mL adapter matching the instrument and immersed in liquid nitrogen for rapid freezing for 5 min. The centrifuge tube was taken out and thawed at room temperature, placed into the 2 mL adapter again, installed into the tissue grinder and ground at 55 Hz for 2 min. Step 3 was repeated twice. The centrifuge tube was taken out and centrifuged for 10 min at 12,000 rpm and 4 °C. The supernatant was collected, transferred to a new 2 mL centrifuge tube, concentrated and dried. An amount of 300 µL acetonitrile:2-amino-3-(2-chloro-phenyl)-propionic acid (4 ppm) solution prepared with 0.1% formic acid (1:9, V/V) (stored at 4 °C) was accurately added to redissolve the sample, the supernatant was filtered using a 0.22 µm membrane and transferred into the detection bottle for LC-MS.

#### Liquid chromatography conditions

The LC analysis was performed on an ACQUITY UPLC System (Waters, Milford, MA, USA). Chromatography was carried out using an ACQUITY UPLC ® HSS T3 (150 × 2.1 mm, 1.8 µm) (Waters, Milford, MA, USA). The column was maintained at 40 °C. The flow rate and injection volume were set at 0.25 mL/min and 2 μL, respectively. As regards LC-ESI (+)-MS analysis, the mobile phases consisted of (C) 0.1% formic acid in acetonitrile (v/v) and (D) 0.1% formic acid in water (v/v). Separation was conducted under the following gradients: 0~1 min, 2% C; 1~9 min, 2~50% C; 9~12 min, 50~98% C; 12~13.5 min, 98% C; 13.5~14 min, 98%~2% C; 14~20 min, 2% C. As regards LC-ESI (-)-MS analysis, the analytes were measured with (A) acetonitrile and (B) ammonium formate (5 mM). Separation was conducted using the following gradients: 0~1 min, 2% A; 1~9 min, 2%~50% A; 9~12 min, 50~98% A; 12~13.5 min, 98% A; 13.5~14 min, 98~2% A; 14~17 min, 2% A [23].

#### Mass spectrum conditions

Mass spectrometric detection of metabolites was performed on Q Exactive (Thermo Fisher Scientific, USA) with an ESI ion source. Simultaneous MS1 and MS/MS (Full MS-ddMS2 mode, data-dependent MS/MS) acquisition was used. The parameters were: sheath gas pressure, 30 arb; aux gas flow, 10 arb; spray voltage, 3.50 kV and -2.50 kV for ESI(+) and ESI(−), respectively; capillary temperature, 325 °C; MS1 range, *m*/*z* 81–1000; MS1 resolving power, 70000 FWHM; the number of data-dependent scans per cycle, 10; MS/MS resolving power, 17500 FWHM; normalized collision energy, 30%; dynamic exclusion time, automatic [24].

### Statistical analysis

Statistical analysis was performed using GraphPad Prism 8.0 software (GraphPad Soft, San Diego, CA, USA). The two groups were compared using Student’s *t*-test, while multiple groups were compared using the one-way analysis of variance (one-way ANOVA). All in vitro experiments were performed three independent times unless otherwise stated. The results were represented as mean ± standard deviation (SD). A value of *P* ≤ 0.05 was considered statistically significant.

## Results

### Effect of p53 mediated by miR-122-5p in A549 cells

**Fig. 1:**
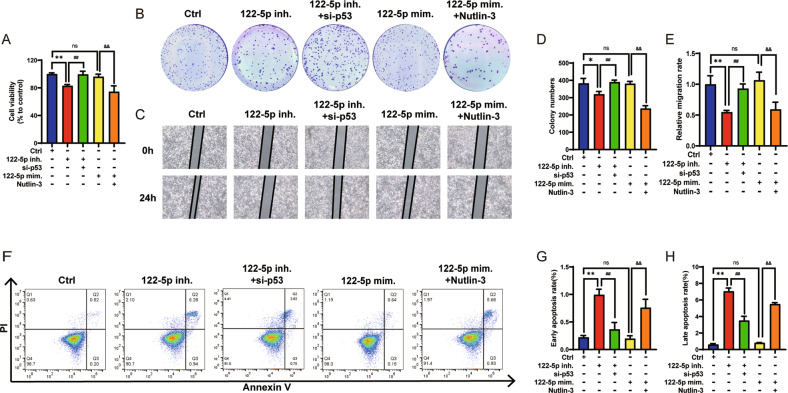
Characterization of miR-122-5p mediated p53 effects in A549 cells. **A** The proliferation of A549 cells treated with 122-5p inh. combined with si-p53 or 122-5p inh. alone or 122-5p mim. combined with Nutlin-3 or 122-5p mim. alone using the CCK-8 assay. The colony formation assay could detect the long-term proliferation ability of A549 cells treated with 122-5p inh. combined with si-p53 or 122-5p inh. alone or 122-5p mim. combined with Nutlin-3 or 122-5p mim. alone (**B**) and the bar graph corresponding to B (**D**). The migration ability of A549 cells treated with 122-5p inh. combined with si-p53 or 122-5p inh. alone or 122-5p mim. combined with Nutlin-3 or 122-5p mim. alone using the cell scratch assay (**C**) and the bar graph corresponding to C (**E**). The apoptosis of A549 cells treated with 122-5p inh. combined with si-p53 or 122-5p inh. alone or 122-5p mim. combined with Nutlin-3 or 122-5p mim. alone using flow cytometry (**F**) and the analysis of early apoptosis (**G**) and late apoptosis (**H**). The above bar graphs were the sum of three independent experiments (mean ± SD). The ctrl group was represented by A549 cells treated using a medium solution containing 122-5p inh. NC and 122-5p mim. NC and si-p53 NC and DMSO, ns: not significant, *P < 0.05 and **P < 0.01 *vs*. Ctrl group, ^#^*P* < 0.05 and ^##^*P* < 0.01 vs. 122-5p inh. group, ^&^*P* < 0.05 and ^&&^*P* < 0.01 vs. 122-5p mim. group.

Previous studies revealed that miR-122-5p interacts with wild-type p53 (wtp53) as a tumor-promoting factor in NSCLC. Therefore, A549 cells could verify the relationship between miR-122-5p and wtp53 in NSCLC cells. The expression of miR-122-5p and p53 in A549 cells and normal lung cells BEAS-2B (Supplementary Figs. S1A and S2A) was compared, indicating that p53 expression was low while that of miR-122-5p was high in A549 cells than in BEAS-2B. Next, A549 cells were treated with a miR-122-5p inhibitor (122-5p inh.), and miR-122-5p mimic (122-5p mim.) and their transfection efficiency (Supplementary Fig. S1B) were evaluated. Different doses of 122-5p inh. and 122-5p mim. were utilized to explore the potential toxicity of BEAS-2B against normal lung cells (Supplementary Fig. S1C, D). A549 cells were also transfected with si-p53 or treated with Nutlin-3, which is a common p53 agonist [25], to assess the effect of different doses of Nutlin-3, and p53 silencing on the proliferation and apoptosis of A549 cells (Supplementary Fig. S2B and S2C–H). si-p53 enhanced the proliferation of A549 cells, while Nutlin-3 inhibited cell proliferation and induced apoptosis, and miR-122-5p was negatively correlated with p53. A549 cells were treated with 122-5p inh., combined with si-p53 or 122-5p inh. alone or 122-5p mim. combined with Nutlin-3 or with 122-5p mim. alone to assess cell proliferation, colony formation, migration, and apoptosis (Fig. 1A–H) and the effect of p53 mediated by miR-122-5p on the phenotype of A549 cells. Meanwhile, this experiment was repeated on another p53 wild-type NSCLC cell line H1944 (Supplementary Fig. S3A–H). The results indicated significant cytotoxicity when miR-122-5p mim. was given to BEAS-2B cells at 8 times dose (*n* = 3, *P* < 0.05). 122-5p inh. significantly reduced cell proliferation, colony formation, and migration of A549 cells and induced cell apoptosis (*n* = 3, *P* < 0.01 or *P* < 0.05), reversed by the transfection with si-p53 (*n* = 3, *P* < 0.01). However, 122-5p mim. had no significant effect on the proliferation, colony formation, migration, and apoptosis of A549 cells. The addition of Nutlin-3 caused a significant inhibition in the proliferation and migration of A549, while apoptosis increased (*n* = 3, *P* < 0.01). These results in H1944 cells were consistent with A549 cells, indicating that miR-122-5p had opposite effects to p53. Thus, p53 reversed cell proliferation, migration and apoptosis affected by miR-122-5p.

### Effect of p53 on the MVA pathway in A549 cells

**Fig. 2:**
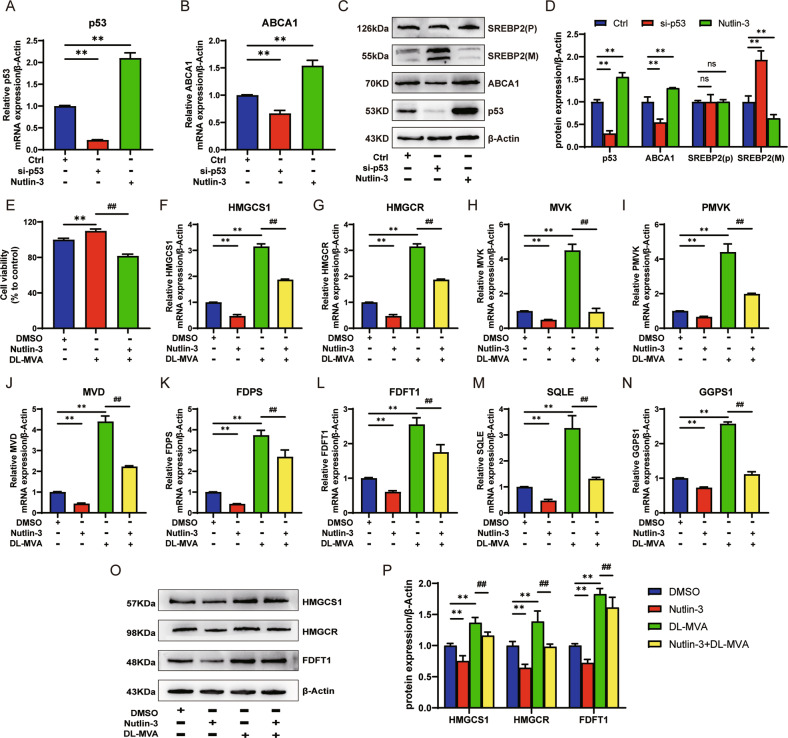
Effect of p53 on the MVA pathway in A549 cells. A549 cells treated with si-p53 or Nutlin-3, RT-qPCR could detect the relative mRNA expression of p53 (**A**) and ABCA1 (**B**). p53, ABCA1, SREBP2(P), SREBP2(M) protein expression using western blotting (**C**) and the bar graph corresponding to C after statistical analysis (**D**) in A549 cells treated with si-p53 or Nutlin-3. **E** The relative mRNA expression of the MVA pathway genes HMGCS1 (**F**), HMGCR (**G**), MVK (**H**), PMVK (**I**), MVD (**J**), FDPS (**K**), FDFT1 (**L**), SQLE (**M**), and GGPS1 (**N**) by RT-qPCR in A549 cells treated with Nutlin-3 combined with DL-MVA or Nutlin-3 alone or DL-MVA alone. The expression of the MVA pathway key proteins HMGCS1, HMGCR, and FDFT1 using western blotting (**O**) and the bar graph corresponding to O (**P**) in A549 cells treated with Nutlin-3 combined with DL-MVA or Nutlin-3 alone or DL-MVA alone. The above bar graphs were the sum of 3 independent experiments (mean ± SD). The ctrl group was A549 cells treated with a medium solution containing si-p53 NC and DMSO, ns: not significant, *P < 0.05 and **P < 0.01 vs. Ctrl or DMSO group, ^#^*P* < 0.05 and ^##^*P* < 0.01 *vs*. DL-MVA group.

The si-p53 and Nutlin-3 effects on the genes and proteins p53, ABCA1, and SREBP2 of the MVA pathway were explored in A549 cells (Fig. 2A–D). si-p53 significantly decreased the expression of p53 and ABCA1 genes and proteins and increased the expression of mature (M) SREBP2 (*n* = 3, *P* < 0.01) but did not affect the precursor (P) SREBP2. Nutlin-3 elevated the expression of p53 and ABCA1 gene and protein (*n* = 3, *P* < 0.01) but did not affect the protein precursor of SREBP2, and it decreased the mature SREBP2 expression (*n* = 3, *P* < 0.01). Nutlin-3 reduced the proliferation of A549 cells upregulated by DL-Mevalonolactone (DL-MVA) (*n* = 3, *P* < 0.01) (Fig. 2E), indicating that p53 affected the MVA pathway in A549 cells. According to previous studies, nine primary genes involved in the regulation, including HMGCS1, HMGCR, MVK, PMVK, MVD, FDPS, FDFT1, SQLE, and GGPS1 of the MVA pathway, were identified [26]. A549 cells were treated with Nutlin-3 combined with DL-MVA or Nutlin-3 alone or DL-MVA alone (Fig. 2F–N). The results revealed that Nutlin-3 inhibited the expression of all nine genes of the MVA pathway (*n* = 3, *P* < 0.01) and blocked the expression of the MVA pathway genes which DL-MVA up-regulates (*n* = 3, *P* < 0.01). Subsequently, the changes in the protein expression of three essential genes, including HMGCS1, HMGCR, and FDFT1 in the MVA pathway were assessed (Fig. 2O–P), and their change trend was consistent with the gene expression (*n* = 3, *P* < 0.01).

### Effect of miR-122-5p on the MVA pathway by targeting p53

**Fig. 3:**
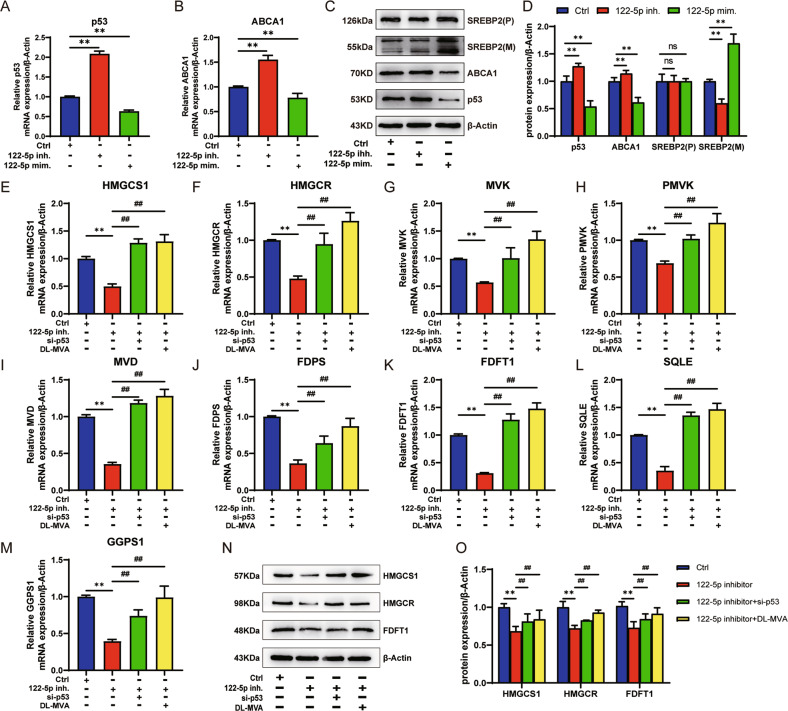
Effect of miR-122-5p on the regulation of the MVA pathway by targeting p53. A549 cells treated with 122-5p inh. or 122-5p mim. and the relative mRNA expression of p53 (**A**) and ABCA1 (**B**) by RT-qPCR. p53, ABCA1, SREBP2(P), and SREBP2(M) protein expression using western blotting (**C**) and the bar graph corresponding to C after statistical analysis (**D**) in A549 cells treated with 122-5p inh. or 122-5p mim. and relative mRNA expression of the MVA pathway genes HMGCS1(**E**), HMGCR (**F**), MVK (**G**), PMVK (**H**), MVD (**I**), FDPS (**J**), FDFT1 (**K**), SQLE (**L**), and GGPS1 (**M**) by RT-qPCR in A549 cells treated with 122-5p inh. combined with si-p53 or 122-5p inh. combined with DL-MVA or 122-5p inh. alone. The expression of the MVA pathway key proteins HMGCS1, HMGCR, and FDFT1 using western blotting (**N**) and the bar graph corresponding to N (**O**) in A549 cells treated with 122-5p inh. combined with si-p53 or 122-5p inh. alone. The above bar graphs were the sum of three independent experiments (mean ± SD). The ctrl group was A549 cells treated with a medium solution containing 122-5p inh. NC and si-p53 NC and DMSO, ns not significant, *P < 0.05 and **P < 0.01 *vs*. Ctrl group, ^#^*P* < 0.05 and ^##^*P* < 0.01 vs. 122-5p inh. group.

A549 cells treated with 122-5p inh., si-p53/122-5p mim. and Nutlin-3 were consistent with the regulation of the MVA pathway; indeed, 122-5p inh. up-regulated the expression of p53 and ABCA1 gene and protein, decreased the expression of mature SREBP2 (*n* = 3, *P* < 0.01), and did not affect the precursor of SREBP2. 122-5p mim. reduced the expression of p53 and ABCA1 gene and protein (*n* = 3, *P* < 0.01) without affecting the protein precursor of SREBP2 while elevating the expression of mature SREBP2 (*n* = 3, *P* < 0.01) (Fig. 3A–D). Therefore, combined with those in Fig. 1, revealed that 122-5p inh. and si-p53 were mutually antagonistic. The expression of the MVA pathway nine genes (Fig. 3E–M) and three key proteins (Fig. 3N, O) of the MVA pathway were examined by adding si-p53 or DL-MVA to 122-5p inh. treated A549. The results showed that si-p53 or DL-MVA restored the expression of the downregulated nine genes and three crucial proteins of the MVA pathway in A549 cells by miR-122-5p (*n* = 3, *P* < 0.01).

### Effect of miR-122-5p on the production of the MVA pathway metabolites through p53

**Fig. 4:**
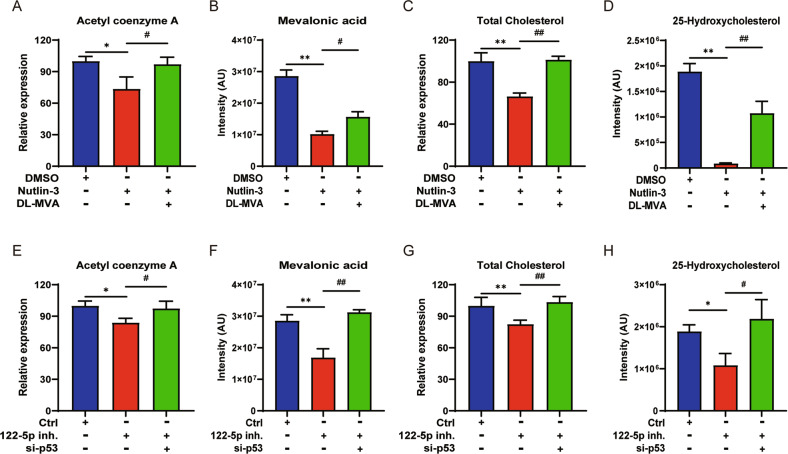
Effect of miR-122-5p on the production of the MVA pathway metabolites through p53. **A** The changes of A-CoA in A549 cells treated with Nutlin-3 combined with DL-MVA or Nutlin-3 alone by ELISA kit. Nutlin-3 combined with DL-MVA or Nutlin-3 alone changes mevalonic acid (**B**) and 25-Hydroxyergosterol (**D**) by LC-MS in treated A549 cells. **C** The changes of TC in A549 cells treated with Nutlin-3 combined with DL-MVA or Nutlin-3 alone by biochemical kit. (**E**) The changes of A-CoA in A549 cells treated with 122-5p inh. combined si-p53 or 122-5p inh. alone using an ELISA kit. The changes of mevalonic acid (**F**) and 25-hydroxyergosterol (**H**) in A549 cells. **G** The changes of TC in A549 cells treated with 122-5p inh. combined with si-p53 or 122-5p inh alone by biochemical kit. The ctrl group was A549 cells treated with a medium solution containing 122-5p inh. NC and si-p53 NC. The above bar graphs were the sum of three independent experiments (mean ± SD), ns: not significant, *P < 0.05 and **P < 0.01 vs DMSO or Ctrl group, ^#^*P* < 0.05 and ^##^*P* < 0.01 vs Nutlin-3 or 122-5p inh. group.

A549 cells were treated with Nutlin-3 combined with DL-MVA or Nutlin-3 alone or 122-5p inh. combined with si-p53 or 122-5p inh. alone. Liquid chromatography coupled to mass spectrometry (LC–MS) could detect the changes in the metabolites of the MVA pathway acetyl-CoA (A-CoA), mevalonic acid, total cholesterol (TC) and the cholesterol derivative 25-hydroxyergosterol. The results showed that 122-5p inh. and Nutin-3 reduced the contents of the upstream products A-CoA and mevalonic acid and those of the downstream products cholesterol and 25-hydroxyergosterol of the MVA pathway (*n* = 3, *P* < 0.01). The transfection with si-p53 or the treatment with DL-MVA restored the original amount of the metabolites of the MVA pathway (*n* = 3, *P* < 0.01) (Fig. 4A–H). It was observed that the proliferation and migration level of A549 cells decreased and the apoptosis level increased by adding SIM, the inhibitor of the MVA pathway. 122-5p mim. prevented SIM-caused inhibition of the MVA pathway. (Supplementary Fig. 4A–F).

### Correlation of miR-122-5p with p53 expression in NSCLC tumor

**Fig. 5:**
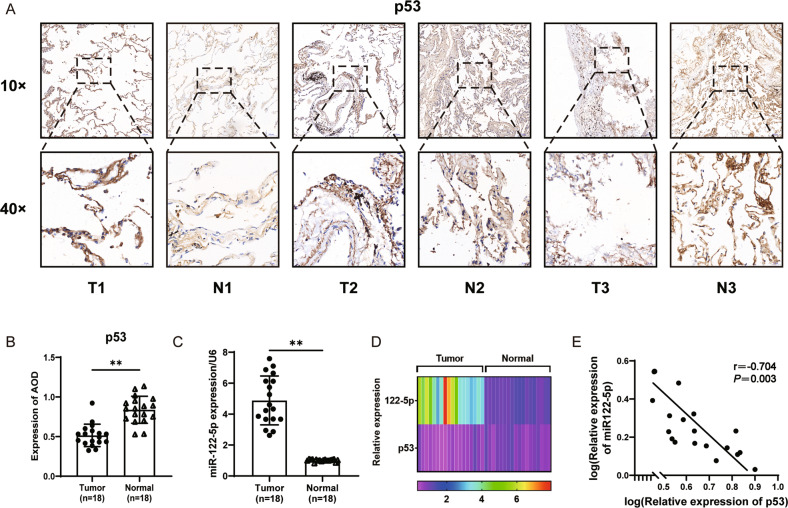
Correlation of MiR-122-5p with p53 expression in NSCLC tumor tissues. p53 in tumor tissues and normal tissues of p53 wild-type NSCLC patients by IHC (**A**) and the bar graph corresponding to A of its relative expression (**B**). **C** miR-122-5p expression in tumor and normal tissues of p53 wild-type NSCLC patients by RT-qPCR. **D** p53 wild-type NSCLC patients with miR-122-5p and p53 relative expression heat map in tumor and normal tissues. **E** The correlation analysis of miR-122-5p and p53 in tumor tissue and normal tissue. The above bar graphs were the sum of three independent experiments (mean ± SD), ns not significant, *P < 0.05 and **P < 0.01.

Tumor (T) and corresponding samples from normal tissues (N) were collected from NSCLC patients to confirm our results, and 18 cases with p53 wild-type were selected based on the case reports and patient information table (Supplementary Table. S1). Samples were cut into sections and stained using HE (Supplementary Fig. S3A) and p53 with immunohistochemistry (IHC) (Fig. 5A). The p53 expression was quantified by analyzing the average optical density (AOD) of the positive cells in specific fields of the slices. The results indicated that the p53 expression in tumor tissues was significantly lower than that in the corresponding normal tissues (Fig. 5B). In contrast, the miR-122-5p expression in the tumor was significantly higher than in the normal tissues (*n* = 18, *P* < 0.01) (Fig. 5C). In addition, miR-122-5p was negatively associated with p53 in NSCLC (*r* = −0.704, *P* = 0.003) (Fig. 5D, E).

### Correlation of NSCLC malignancy with the expression of key genes in the MVA pathway

**Fig. 6:**
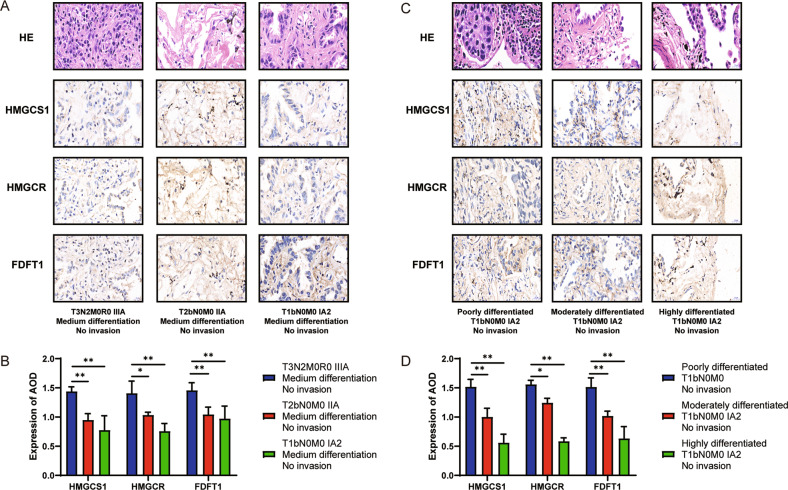
Correlation of malignant NSCLC with the expression of key genes in the MVA pathway. HMGCS1, HMGCR, and FDFT1 protein expression in tumor tissues of p53 wild-type NSCLC patients with different cancer stages by IHC staining (**A**) (×40 magnification) and the bar graph corresponding to A of their expression (**B**). p53 wild-type NSCLC cancers with different degrees of differentiation by HE, HMGCS1, HMGCR, and FDFT1 by IHC staining (**C**) (×40 magnification) and the bar graphs of their expression (**D**). AOD values were measured from positive areas of three randomly selected different fields of view (mean ± SD), ns not significant, *P < 0.05 and **P < 0.01.

Our study found that the expression of the MVA pathway key genes in the tumors of NSCLC patients was not always strictly higher than that in corresponding normal tissues (Supplementary Fig. S5B). Similar results could be found in A549 and BEAS-2B cells (Supplementary Fig. S5C). However, NSCLC patients with a high expression of the key genes HMGCS1, HMGCR and FDFT1 of the MVA pathway had a poor survival rate, depicted by the TCGA database (Supplementary Fig. S5D–F). Moreover, the expression of the above key genes in the MVA pathway varied widely among tumors from different patients. Among the selected 18 samples with p53 wild-type NSCLC, six samples without tissue invasion were chosen based on the NSCLC tumor stage and the degree of differentiation of the tumor tissue. The expression of HMGCS1, HMGCR, and FDFT1 were compared among them depending on the IHC results (Fig. 6A, C). The results showed that the later the tumor stage, the higher the expression of the key genes in the MVA pathway under the same degree of differentiation and no invasion (*n* = 3, *P* < 0.01) (Fig. 6B). Moreover, the lower the degree of differentiation, the higher the expression of the above key genes of the MVA pathway under the same tumor stage and no invasion (*n* = 3, *P* < 0.01) (Fig. 6D). The later tumor stage and the lower the differentiation degree are often the pathological manifestations of more severe NSCLC. The above results showed that the high gene expression in the MVA pathway was associated with more severe NSCLC progression.

## Discussion

This study revealed that 122-5p inh. inhibited the proliferation and migration of A549 cells and promoted apoptosis. It was reversed by adding si-p53. 122-5p mim. had no evident effect on A549 characterization, which could be related to the long-term high expression of miR-122-5p cancer cells. The addition of Nutliln-3 reduced the proliferation and migration of cells, while the apoptosis level increased. These phenomena indicated that p53 was regulated by miR-122-5p. Furthermore, the correlation between miR-122-5p and p53 in clinical samples, combined with the analysis of the luciferase experimental report in the previous study [7], revealed that p53 was the direct regulatory target of miR-122-5p. Previous studies showed that some proteins, including α-smooth muscle actin [27] and hypoxia-inducible factor-1 [28] could be the active sites of miR-122-5p. Our study provided a new research idea for the applying of miR-122-5p. Specific miRNAs could be used as biomarkers for a high-sensible and non-invasive diagnosis of NSCLC at an early-stage [29]. miR-122-5p is highly expressed in various tumors, including renal cell carcinoma, ovarian cancer, liver cancer, and pancreatic cancer [30–33]. Thus, it could play a clinical role as a cancer marker in the future [34, 35].

p53 protein has a research history of nearly 50 years and has the gene with the highest correlation with human tumors found so far [36]. Its role in cell metabolism and various aspects of tumor suppression still has great research value. p53 affects tumor growth and reproduction by regulating lipid metabolism, but the regulatory effect of different p53 forms on the MVA pathway are diametrically opposite. Wtp53 inhibits tumor growth by downregulating the MVA pathway [12], whereas mutant p53 increases the MVA pathway signaling to promote tumor proliferation, metastasis, and tumor drug resistance [37]. This study primarily focused on the regulation of wtp53 on the MVA pathway in NSCLC cells. The expression of p53 and ABCA1 decreased in A549 cells treated with si-p53, the expression of mature SREBP2 increased, and the MVA pathway was activated. Nutlin-3 treatment indicated completely opposite results, while the addition of DL-MVA reversed the expression of genes in the MVA pathway, indicating that p53 was involved in the regulation of the MVA pathway in NSCLC. The MVA pathway regulation by p53 has multiple action sites [38, 39]. Our research established that ABCA1 is one of the targets of p53 regulating the MVA pathway in NSCLC. However, whether the MVA pathway is regulated by other p53 target genes, including LPIN1, and plays a role in NSCLC needs further study.

The MVA pathway is closely related to the occurrence and development of cancer [40]. Cholesterol-like metabolites produced by the MVA pathway can not only provide raw materials for the synthesis of the membranes of cancer cells but also regulate the function of glycosphingolipids on the surface of cancer cells to limit the immune recognition of tumors by the body [41–44]. The MVA signaling pathway regulates protein N-glycosylation associated with tumor metastasis [45].

Previous studies revealed that the genes in the MVA pathway are often positively correlated with the development of tumors, and their expression in tumors is higher than in the corresponding normal tissues most of time [46, 47]. However, this general rule disappeared when we analyzed the expression of key genes in the MVA pathway in NSCLC. We hypothesized that it could be related to the environment in which the lungs are located. Metabolic reprogramming and lipid metabolism of tumor cells are altered in a hypoxic microenvironment [48, 49]. A study showed that the mRNA and protein expression of SREBP1 is increased in A549 cells under hypoxic conditions [50]. Another research indicates that hyperbaric oxygen therapy reduces triglyceride and TC levels and improves insulin sensitivity in obese rats [51]. A recent study showed that intermittent hypoxic-hyper oxygen therapy improves blood lipids and anti-inflammatory status in patients with metabolic syndrome [52]. Several studies have indicated that the MVA pathway can be modulated by the hypoxia-inducible factor 1α (HIF-1α) [53, 54]. HIF-1α is the primary regulator of hypoxia response, inhibiting the tumor cells migration and preventing cancer progression [55]. Research shows that HIF-1α can reduce the level of miR-122 under hypoxia [56]. We use HIF-1α activity inhibitor 2-MeOE2 alone or 2-MeOE2 combined with 122-5p mim. further studied on A549 cells. By detecting A549 cell proliferation, migration, apoptosis and the MVA pathway key genes (Supplementary Fig. 6A–I), HIF-1α may play a role in the MVA pathway regulated by miR-122-5p, which also preliminarily proves our conjecture. Lung tissue is the main site for gas exchange, and its special oxygen-containing environment may affect the MVA pathway. Future in-depth exploration of lipid metabolism in the lungs could be an important study for the further evaluation of their role in the lungs.

Although the MVA pathway genes are not strictly highly expressed in NSCLC, our study revealed that the high expression of the MVA pathway genes was associated with more severe cancer lesions. Thus, targeting the MVA pathway could be a promising therapeutic strategy in cancer treatment [57]. At present, anti-tumor drugs targeting the MVA pathway can be roughly divided into three categories according to their targets: anti-HMGCR, anti-FDPS and anti-SREBP2 [58–61]. The former two are represented by statins and bisphosphonates and achieved good therapeutic results [62, 63] while targeting SREBP2 to treat cancer has broader research prospects. SREBP2 inhibitors can be roughly divided into hydrobromide, microRNA and natural medicine. Hydrobromide prevents the transport of the sterol regulatory element-binding protein cleavage-activating protein from the endoplasmic reticulum to the Golgi apparatus, thereby inhibiting the activation of SREBP2 [64]. A study showed that miR-130b significantly affects cholesterol production mediated by SREBP2 [65], and miR-33a targets SREBP2 to block the invasion and metastasis of NSCLC cells [66]. Some drugs, including artesunate and emodin, target SREBP2 to regulate lipid metabolism related to the MVA pathway and inhibit tumor growth [67, 68]. Our recent research shows that berberine hydrochloride, the active ingredient in Coptis chinensis, has a good docking activity with SREBP2. Thus, it exerts a tumor suppressor effect by affecting the expression of the MVA pathway genes [26]. Natural medicines may affect microRNAs and, consequently, SREBP2. For example, resveratrol affects non-alcoholic fatty liver disease in rats by targeting miR-34a to regulate SREBP2 [69]. Salidroside inhibits insulin resistance by downregulating miR-21, which regulates SREBP2 [70]. Our discovery of the involvement of the miR-122-5p/p53/MVA axis in the development of NSCLC, the prevention and treatment of tumors by regulating the MVA pathway through miRNA could be more widely used in the future.

**Fig. 7:**
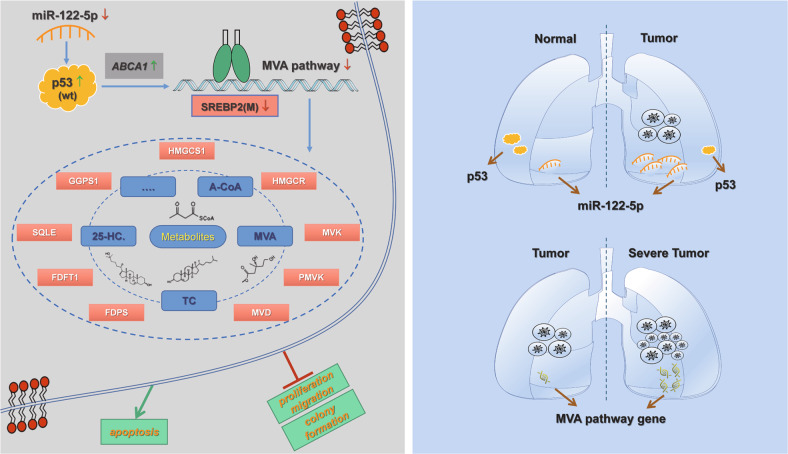
Mechanism diagram of miR-122-5p regulates the mevalonate pathway by targeting p53 in non-small cell lung cancer. Inhibition of miR-122-5p in NSCLC cells A549 led to the up-regulation of p53 expression. This elevated the expression of the p53 target gene ABCA1, inhibited the maturation of SREBP2, hindered the MVA pathway, affected the proliferation and migration of A549 cells, and promoted apoptosis of A549 cells. MiR-122-5p was negatively correlated with p53 expression in NSCLC tumor tissues. The malignancy of NSCLC could be positively associated with the expression of key genes in the MVA pathway.

Our study demonstrated that miR-122-5p was negatively correlated with p53 in NSCLC, acting as a tumor-promoting factor in NSCLC cells by inhibiting the p53-regulated the MVA pathway. The interference of the expression of miR-122-5p leads to changes in cell proliferation, migration, and apoptosis, which in turn affects tumor development (Fig. 7). Our results enrich the theoretical basis for considering miRNAs in treating of NSCLC and provide a foundation for the future development of targeted drugs.

## Supplementary information

Supplemental Figures and Tables

Western blot raw data

aj-checklist

## Data Availability

Raw data have been placed in the supporting file “Western blot raw data”. The datasets generated during and/or analyzed during the current study are available from the corresponding author on reasonable request.
