# Supplementary material for: MiR-122-5p regulates the mevalonate pathway by targeting p53 in non-small cell lung cancer
Source: Cell Death Dis. 2023 Apr 1;14(4):234. doi: 10.1038/s41419-023-05761-9 (PMC10067850; doi:10.1038/s41419-023-05761-9)
Supplement: Supplementary file 1 — Supplemental Figures and Tables [file 41419_2023_5761_MOESM1_ESM.docx]

MiR-122-5p regulates the mevalonate pathway by targeting p53 in non-small cell lung cancer

Yu-kun Zheng^1,2#^, Zhong-shi Zhou^2,3#^, Guang-zhong Wang^2,3#^, Ji-yuan Tu^2,3^, Huan-bo Cheng^2^, Shang-zhi Ma^2^, Chang Ke^2^, Yan Wang^2^, Qi-pan Jian^2^, Yu-hang Shu^2^, Xiao-wei Wu^1^*****.

**Affiliation:**

^1^Department of Thoracic Surgery, Tongji Hospital, Tongji Medical College, Huazhong University of Science and Technology, Wuhan 430030, China.

^2^College of Pharmacy, Hubei University of Chinese Medicine, Wuhan 430065, China.

^3^Hubei Engineering Technology Research Center of Chinese Material Medical Processing Technology, Wuhan 430065, China.

^#^These authors contributed equally: Yu-kun Zheng, Zhong-shi Zhou and Guang-zhong Wang.

***Corresponding author:**

Xiao-wei Wu, Chief Physician

No. 1095 Jiefang Avenue, Qiaokou District, Wuhan, Hubei Province, China

E-mail: wuxiaowei119@hust.edu.cn

Tel: (8627)83662688

**Supple****mentary Figures**

**Supplementary Figure 1:** Effect of miR-122-5p in lung cells.

**Supplementary Figure 2:** Effect of p53 in A549 cells.

**Supplementary Figure 3:** Characterization of miR-122-5p mediated p53 effects in H1944 cells.

**Supplementary Figure 4:** Effect of SIM in A549 cells.

**Supplementary Figure 5:** Analysis of the MVA pathway genes in NSCLC.

**Supplementary Figure 6:** Effect of 2-MeO2 in A549 cells.

**Supplementary Tables**

**Supplementary Table 1:** Patient Information Collection Form.

**Supplementary Table 2:** The primer sequences of miR-122-5p inhibitor, miR-122-5p mimic, and si-p53.

**Supplementary Table 3:** The primer sequences of the nine genes from the MVA pathway.

**
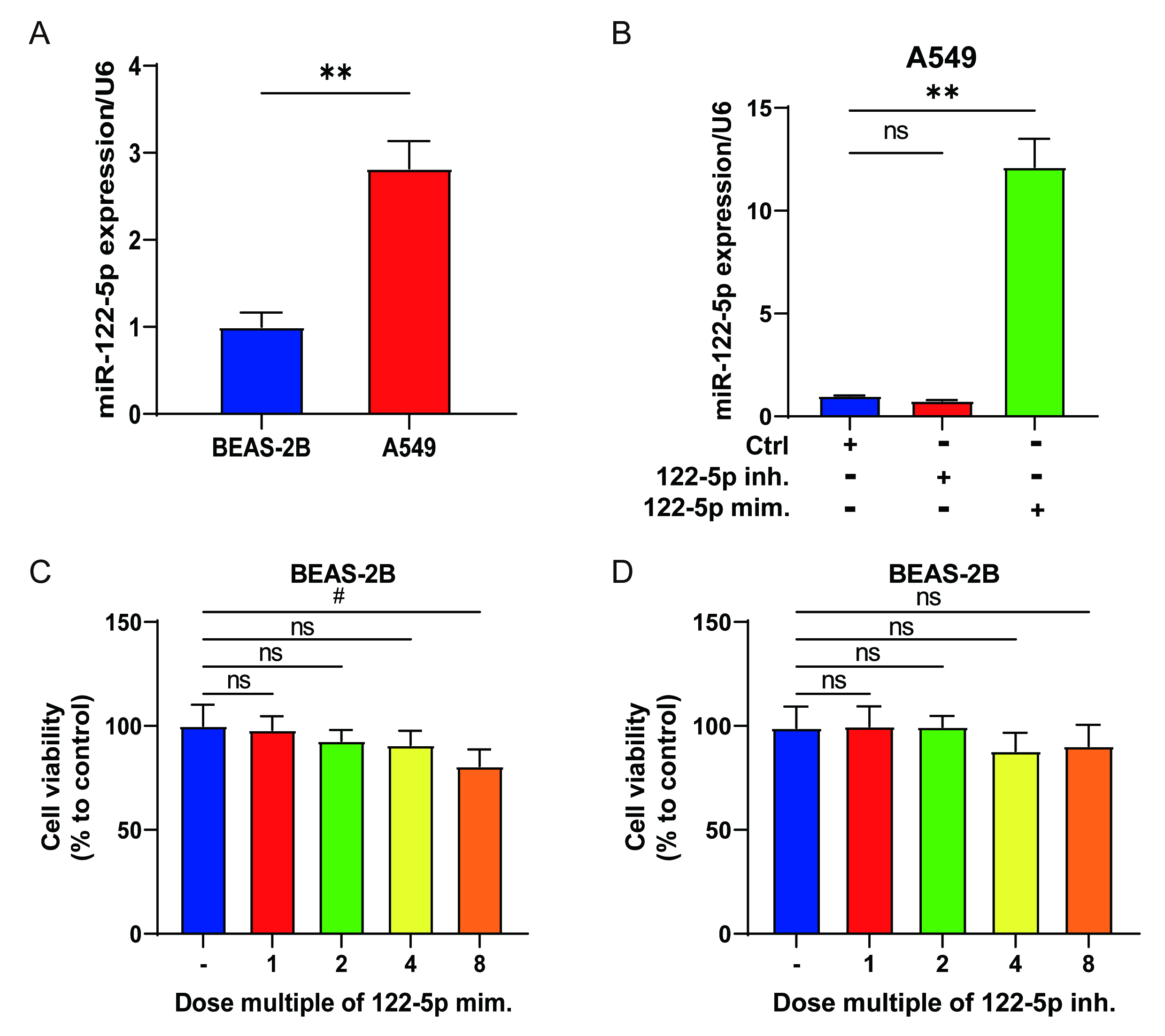
**

**Supplementary Figure 1: Effect of miR-122-5p in lung cells.** (A) Relative expression of miR-122-5p in BEAS-2B cells and A549 cells by RT-qPCR. (B) Relative miR-122-5p expression in A549 cells treated with 122-5p inh. or 122-5p mim., by RT-qPCR. (C) The effect of different doses of 122-5p mim. on the BEAS-2B cell survival. (D) The effect of different doses of 122-5p inh. on the BEAS-2B cell survival. 122-5p inh. relies on blocking the binding of miR-122-5p to downstream sites. Thus, the intracellular expression level of miR-122-5p was not decreased. The ctrl group was A549 cells treated with a medium containing 122-5p inh. NC and 122-5p mim. NC. The above bar graphs were the sum of 3 independent experiments (mean ± SD), ns: not significant, **P* < 0.05 and ***P* < 0.01 *vs*. Ctrl. group, ^#^*P* < 0.05 and ^##^*P* < 0.01 *vs*. BEAS-2B no adding group.

**
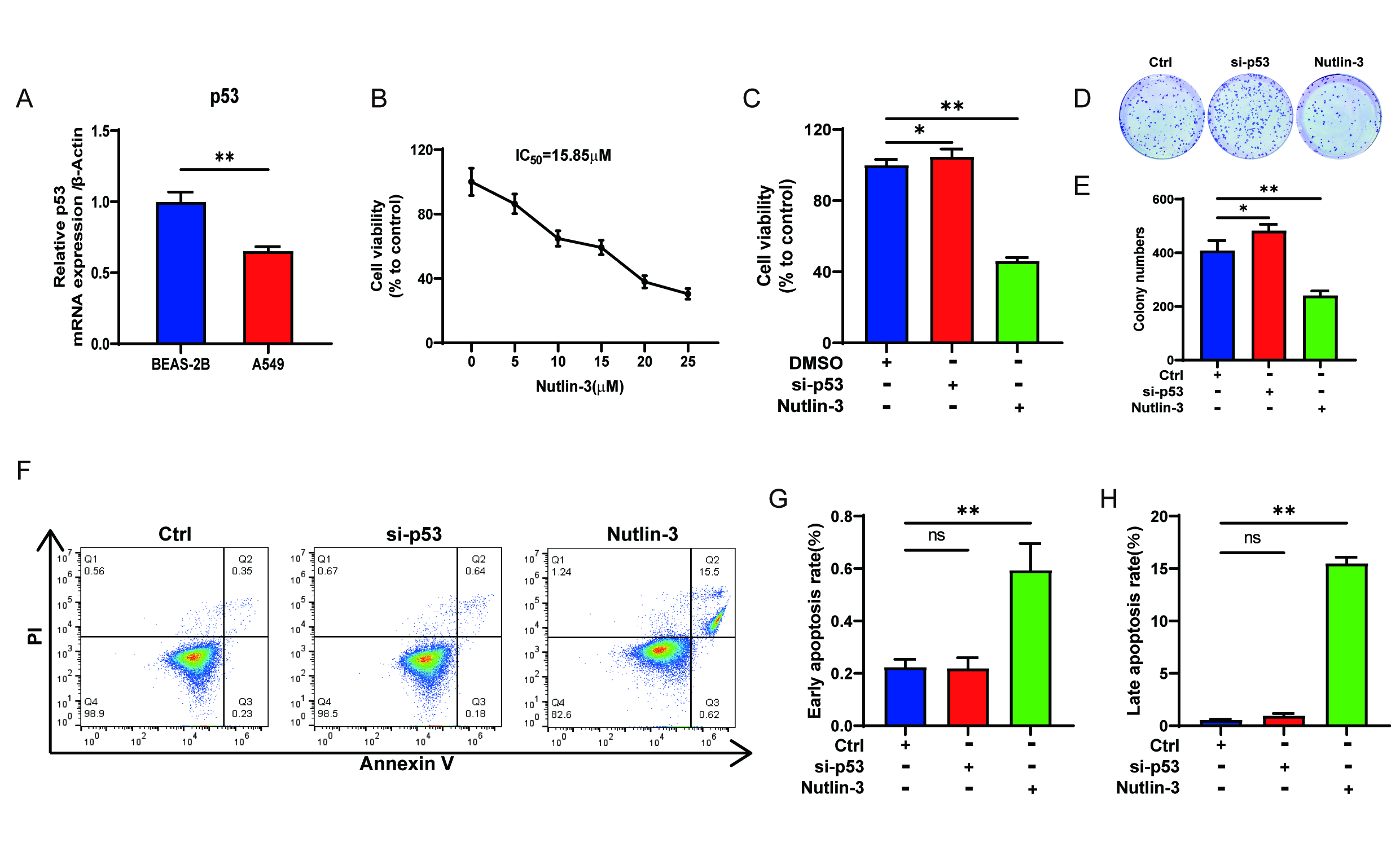
**

**Supplementary Figure 2: Effect of p53 in A549 cells.** (A) p53 expression in BEAS-2B and A549 cells by RT-qPCR. (B) Effect of different doses of Nutlin-3 on the survival of A549 cells. (C) CCK-8 was used to detect the proliferation of A549 cells treated with si-p53 or Nutlin-3. The colony-forming ability of A549 cells treated with si-p53 or Nutlin-3 (D) and its quantification (E). (F) Apoptosis of A549 cells treated with si-p53 or Nutlin-3 was detected using flow cytometry. Early apoptosis (G) and late apoptosis (H) of A549 cells treated with si-p53 or Nutlin-3 levels. The ctrl group was A549 cells treated with a medium containing si-p53 NC and DMSO. The bar graphs were the sum of 3 independent experiments (mean ± SD), ns: not significant, **P* < 0.05 and ***P* < 0.01.

**
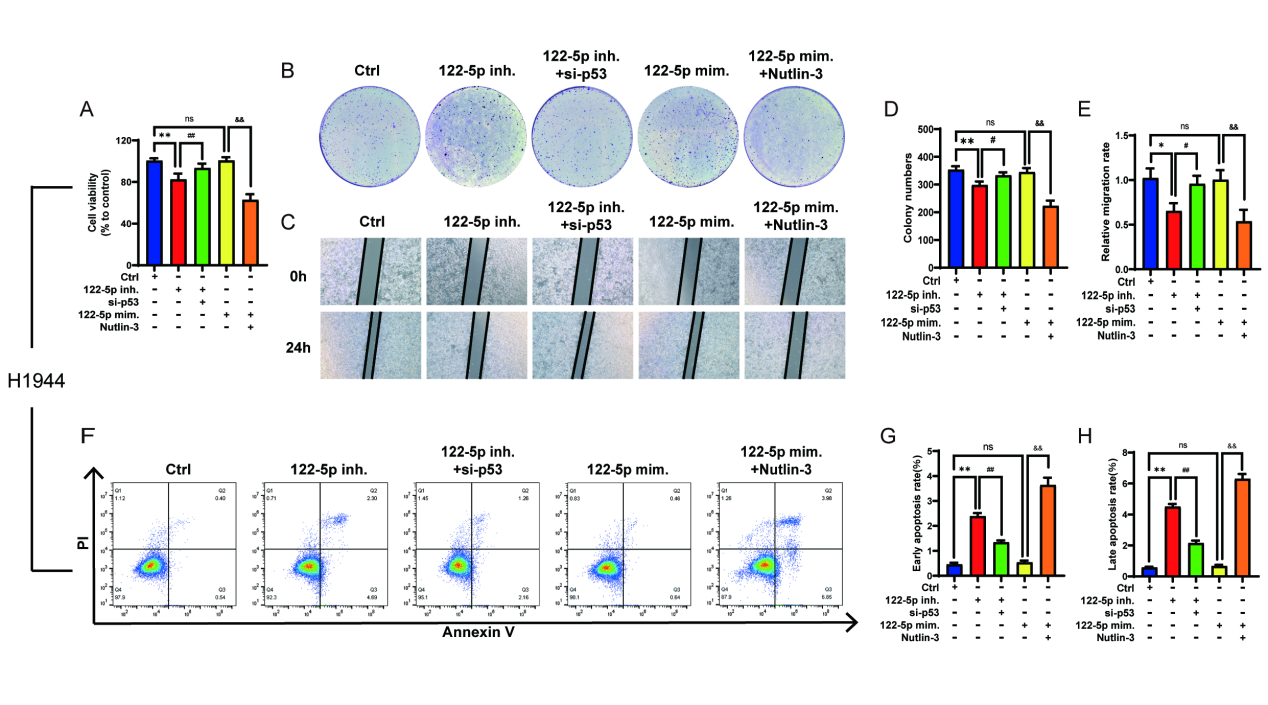
**

**Supplementary Figure 3: Characterization of miR-122-5p mediated p53 effects in H1944 cells.** (A) The proliferation of H1944 cells treated with 122-5p inh. combined with si-p53 or 122-5p inh. alone or 122-5p mim. combined with Nutlin-3 or 122-5p mim. alone using the CCK-8 assay. The colony formation assay could detect the long-term proliferation ability of H1944 cells treated with 122-5p inh. combined with si-p53 or 122-5p inh. alone or 122-5p mim. combined with Nutlin-3 or 122-5p mim. alone (B) and the bar graph corresponding to B (D). The migration ability of H1944 cells treated with 122-5p inh. combined with si-p53 or 122-5p inh. alone or 122-5p mim. combined with Nutlin-3 or 122-5p mim. alone using the cell scratch assay (C) and the bar graph corresponding to C (E). The apoptosis of H1944 cells treated with 122-5p inh. combined with si-p53 or 122-5p inh. alone or 122-5p mim. combined with Nutlin-3 or 122-5p mim. alone using flow cytometry (F) and the analysis of early apoptosis (G) and late apoptosis (H). The above bar graphs were the sum of three independent experiments (mean ± SD). The ctrl group was represented by H1944 cells treated using a medium solution containing 122-5p inh. NC and 122-5p mim. NC and si-p53 NC and DMSO, ns: not significant, **P* < 0.05 and ***P* < 0.01 *vs*. Ctrl group, ^#^*P* < 0.05 and ^##^*P* < 0.01 *vs*. 122-5p inh. group, ^&^*P* < 0.05 and ^&&^*P* < 0.01 *vs*. 122-5p mim. group.

**
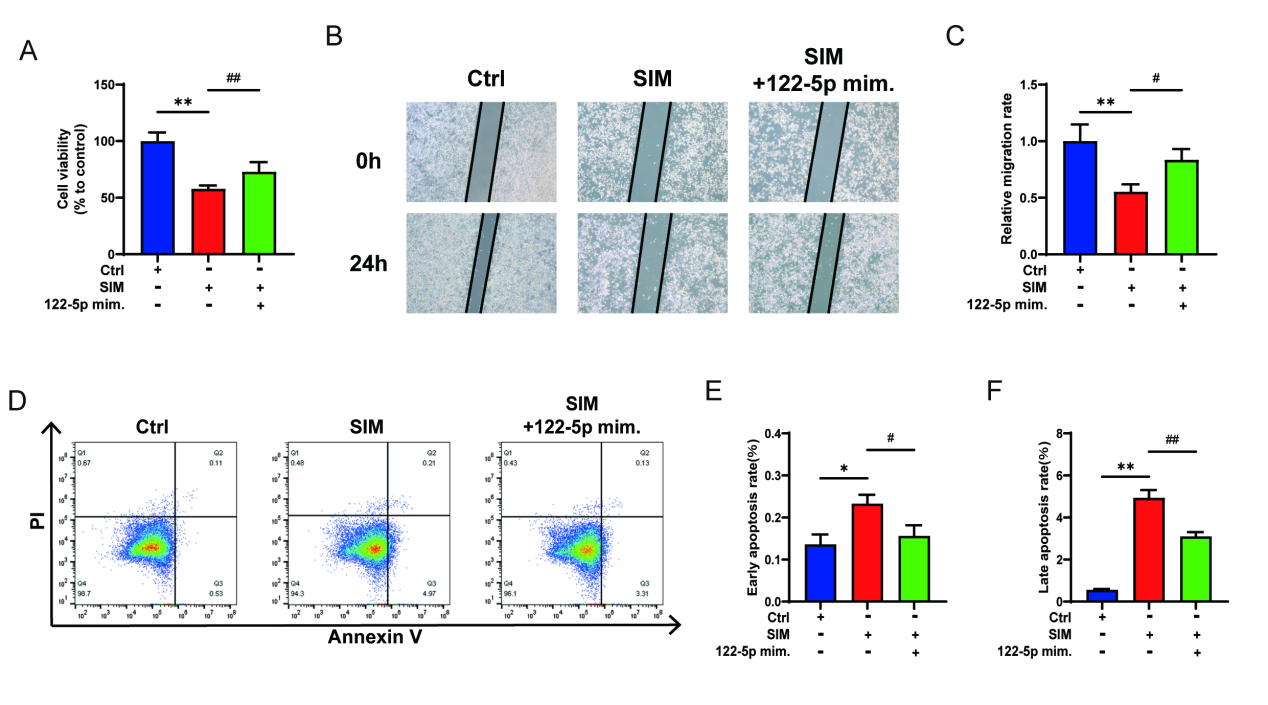
**

**Supplementary Figure 4: Effect of SIM in A549 cells.** (A) The proliferation of A549 cells treated using SIM combined with 122-5p mim. or SIM alone with the CCK-8 assay. The migration ability of A549 cells treated with SIM combined with 122-5p mim. or SIM alone by cell scratch assay (B) and the bar graph corresponding to B (C). The apoptosis of A549 cells treated with SIM combined with 122-5p mim. or SIM alone by flow cytometry (D) and the analysis of early apoptosis (E) and late apoptosis (F). The above bar graphs were the sum of three independent experiments (mean ± SD). The ctrl group was represented by A549 cells treated with a medium solution having 122-5p mim. NC and DMSO, ns: not significant, **P* < 0.05 and ***P* < 0.01 *vs*. Ctrl group, ^#^*P* < 0.05 and ^##^*P* < 0.01 *vs*. SIM group.

**
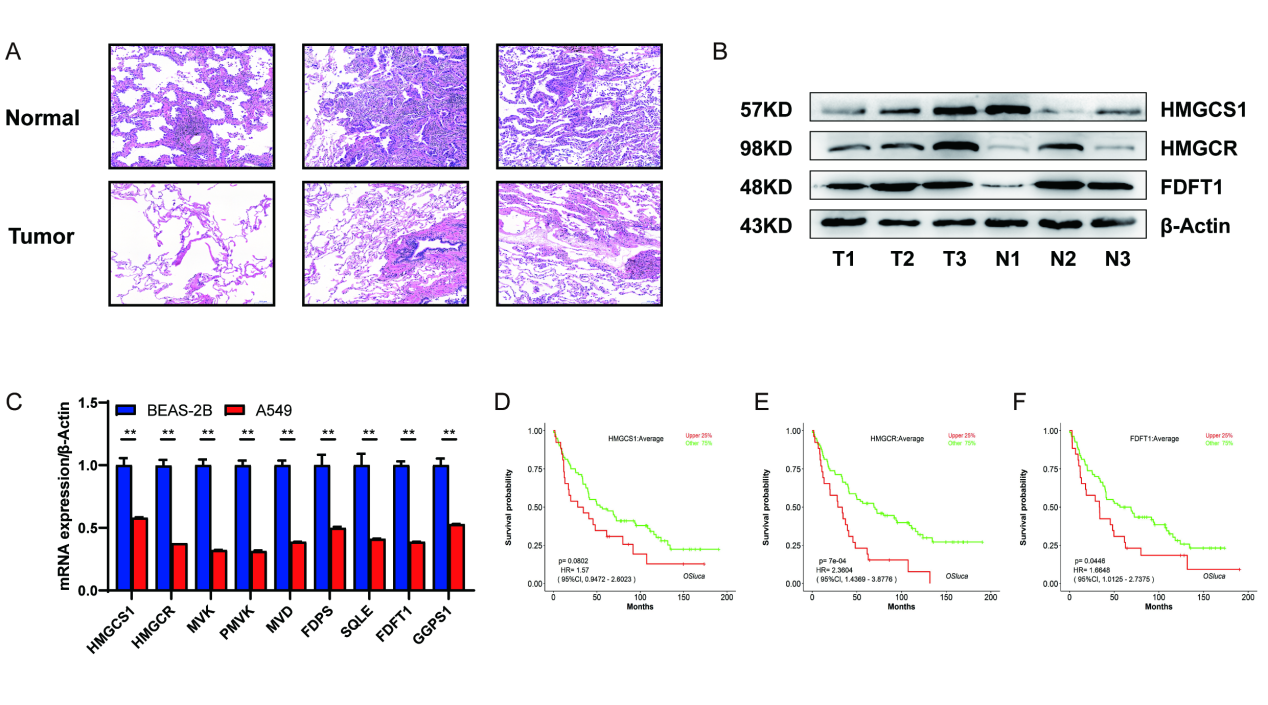
**

**Supplementary Figure 5: Analysis of the MVA pathway genes in NSCLC.** (A) Tumor and normal tissues from NSCLC patients by HE sections (10× magnification). (B) Protein expressions of HMGCS1, HMGCR, and FDFT1 in tumor tissues (T) and normal tissues (N) of NSCLC patients using western blotting. (C) The relative expression of the MVA pathway genes in BEAS-2B cells and A549 cells using RT-qPCR. Survival curves of HMGCS1 (D), HMGCR (E) and FDFT1 (F) in NSCLC patients. Patient survival data were obtained from Biomedical Informatics Institute's database (https://bioinfo.henu.edu.cn/DatabaseList.jsp). Data source: GSE37745; Type: Adenocarcinoma; Survival: OS; Split patients by: Upper 25%. The above data in (C) were the sum of three independent experiments (mean ± SD), ns: not significant, **P* < 0.05 and ***P* < 0.01.

**
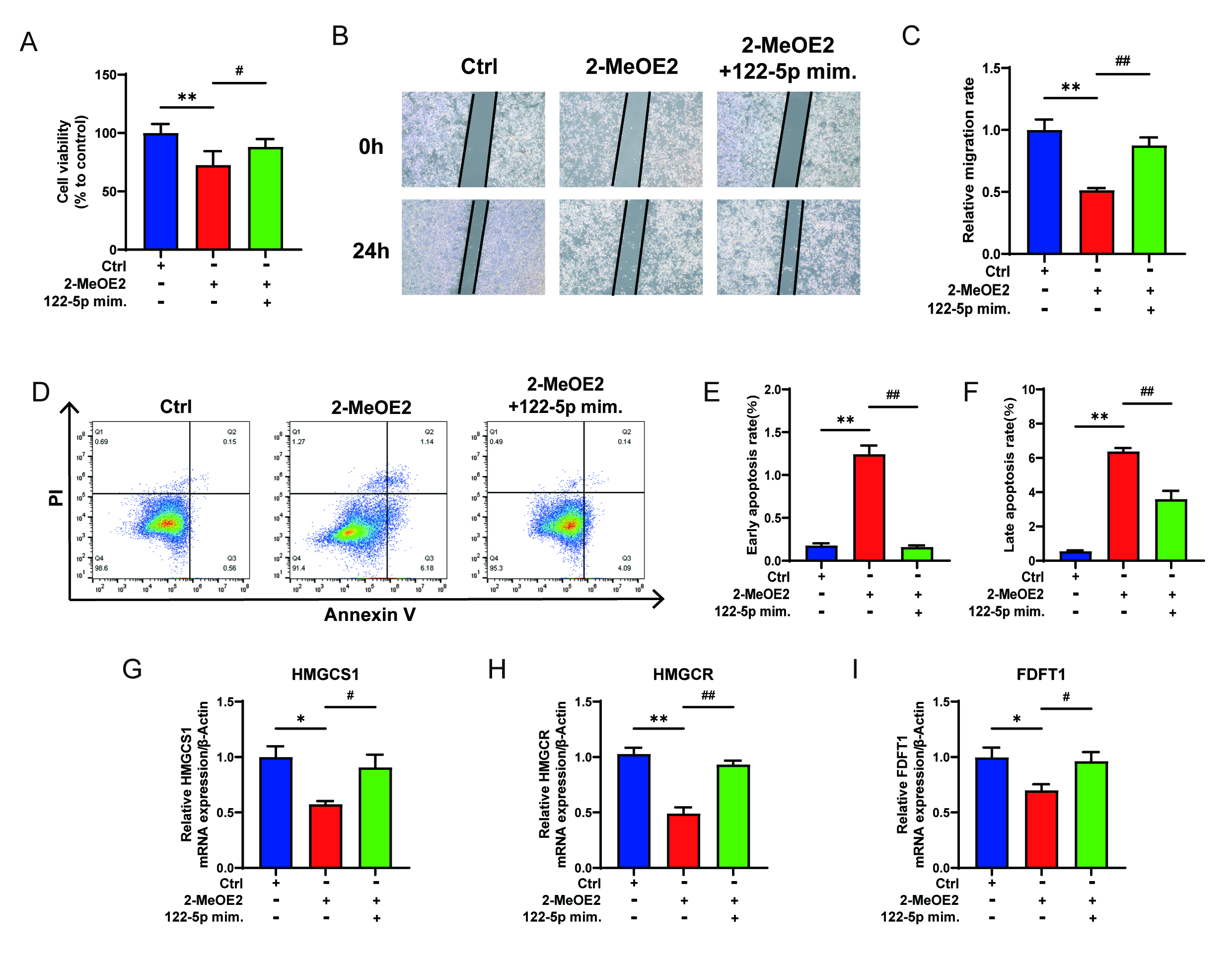
**

**Supplementary Figure 6: Effect of 2-MeO2 in A549 cells.** (A) The proliferation of A549 cells treated using 2-MeOE2 combined with 122-5p mim. or 2-MeOE2 alone with the CCK-8 assay. The migration ability of A549 cells treated with 2-MeOE2 combined with 122-5p mim. or 2-MeOE2 alone by cell scratch assay (B) and the bar graph corresponding to B (C). The apoptosis of A549 cells treated with 2-MeOE2 combined with 122-5p mim. or 2-MeOE2 alone using flow cytometry (D) and the analysis of early apoptosis (E) and late apoptosis (F). A549 cells treated with 2-MeOE2 combined with 122-5p mim. or 2-MeOE2 alone and relative mRNA expression of HMGS1 (G), HMGCR (H) and FDFT1 (I) using RT-qPCR. The above bar graphs were the sum of three independent experiments (mean ± SD). The ctrl group was represented by A549 cells treated with a medium solution having 122-5p mim. NC and DMSO, ns: not significant, **P* < 0.05 and ***P* < 0.01 *vs*. Ctrl group, ^#^*P* < 0.05 and ^##^*P* < 0.01 *vs*. 2-MeOE2 group.

**Supplementary Table 1: Patient Information Collection Form.**

| Number | Age | Height(cm) | Weight(kg) | Gender | Family history of cancer |
| --- | --- | --- | --- | --- | --- |
| #1 | 62 | 168 | 72 | Female | - |
| #2 | 50 | 172 | 59 | Male | - |
| #3 | 44 | 163 | 60 | Female | - |
| #4 | 65 | 172 | 76 | Male | - |
| #5 | 55 | 168 | 65 | Male | lung cancer(Mother) |
| #6 | 63 | 175 | 56 | Male | - |
| #7 | 63 | 173 | 70 | Male | Rectal cancer(Father) |
| #8 | 69 | 153 | 53 | Female | lung cancer(Father) |
| #9 | 63 | 168 | 65 | Male | - |
| #10 | 47 | 153 | 78 | Female | - |
| #11 | 60 | 150 | 45 | Female | - |
| #12 | 61 | 153 | 62 | Female | - |
| #13 | 53 | 163 | 70 | Female | - |
| #14 | 45 | 175 | 75 | Male | - |
| #15 | 45 | 153 | 57 | Female | - |
| #16 | 34 | 175 | 87 | Male | - |
| #17 | 54 | 167 | 70 | Male | - |
| #18 | 47 | 159 | 54 | Female | - |

| Lung  disease history | Smoking status | Smoking  age(years) | Blood  sugar(mmol/L) | Tumor  stage |
| --- | --- | --- | --- | --- |
| - | - | - | 5.30 | T1bN0M0 IA2 |
| - | 20/day | 30 | 5.24 | T1bN0M0 IA2 |
| - | - | - | 5.54 | T1bN0M0 IA2 |
| - | 10/day | 30 | 5.35 | T1bN0M0 IA2 |
| - | 15/day | 30 | 4.79 | T1bN0M0 IA2 |
| - | - | - | 5.19 | T2bN0M0 IIA |
| - | 20/day | 40 | 8.25 | T3N2M0R0 IIIA |
| - | - | - | 6.12 | T3N0M0 IIB |
| - | 20/day | 30 | 4.97 | T1bN0M0 IA2 |
| - | - | - | 5.01 | T1bN0M0 IA2 |
| - | - | - | 4.31 | T2bN0M0 IIA |
| - | 20/day | 30 | 5.44 | T1aN0M0R0 IA1 |
| - | - | - | 5.12 | T1aN0M0R0 IA1 |
| - | - | - | 4.79 | T1aN0M0R0 IA1 |
| - | - | - | 4.7 | T1aN0M0R0 IA1 |
| - | 5/day | 10 | 5.2 | T1aN0M0R0 IA1 |
| - | 15/day | 30 | 5.26 | T1bN0M0 IA2 |
| - | - | - | 4.69 | T1aN0M0R0 IA1 |

**Supplementary Table 2: The primer sequences of miR-122-5p inhibitor, miR-122-5p mimic, and si-p53.**

| **ID** | **Primer** | **Primer sequence(5'to3')** | **Base number** |
| --- | --- | --- | --- |
| 1 | hsa-miR-122-5p inhibitor NC | CAGUACUUUUGUGUAGUACAA | 21 |
| 2 | hsa-miR-122-5p inhibitor | CAAACACCAUUGUCACACUCCA | 22 |
| 3 | hsa-miR-122-5p mimics NC-Sense | UUCUCCGAACGUGUCACGUTT | 21 |
| 4 | hsa-miR-122-5p mimics NC-Antisense | ACGUGACACGUUCGGAGAATT | 21 |
| 5 | hsa-miR-122-5p mimics-Sense | UGGAGUGUGACAAUGGUGUUUG | 22 |
| 6 | hsa-miR-122-5p mimics-Antisense | AACACCAUUGUCACACUCCAUU | 22 |
| 7 | si-p53-Homo-1-Sense | GCAUCUUAUCCGAGUGGAATT | 21 |
| 8 | si-p53-Homo-1-Antisense | UUCCACUCGGAUAAGAUGCTT | 21 |
| 9 | si-p53-Homo-2-Sense | CCCGGACGAUAUUGAACAATT | 21 |
| 10 | si-p53-Homo-2-Antisense | UUGUUCAAUAUCGUCCGGGTT | 21 |
| 11 | si-p53-Homo-3-Sense | CACUACAACUACAUGUGUATT | 21 |
| 12 | si-p53-Homo-3-Antisense | UACACAUGUAGUUGUAGUGTT | 21 |

**Supplementary Table 3: The primer sequences of the nine genes from the MVA pathway.**

| **ID** | **Primer** | **Primer sequence(5'to3')** | **Base number** |
| --- | --- | --- | --- |
| 1 | β-actin-F-Homo | GGATTCCTATGTGGGCGACGA | 21 |
| 2 | β-actin-R-Homo | GCGTACAGGGATAGCACAGC | 20 |
| 3 | P53-F-Homo | ACCTATGGAAACTACTTCCTGAAA | 24 |
| 4 | P53-R-Homo | CTGGCATTCTGGGAGCTTCA | 20 |
| 5 | ABCA1-F-Homo | AGGCTTGTCAAGGGGTAGGA | 20 |
| 6 | ABCA1-R-Homo | GCAGCAGCTGACATGTTTGT | 20 |
| 7 | SREBP2-F-Homo | CCTGGGAGACATCGACGAGAT | 21 |
| 8 | SREBP2-R-Homo | TGAATGACCGTTGCACTGAAG | 21 |
| 9 | HMGCS1-F-Homo | TGTCCTTTCGTGGCTCACTC | 20 |
| 10 | HMGCS1-R-Homo | CTGCAGTCTCCAGGTCTGTC | 20 |
| 11 | HMGCR-F-Homo | TTCGGTGGCCTCTAGTGAGA | 20 |
| 12 | HMGCR-R-Homo | GATGGGAGGCCACAAAGAGG | 20 |
| 13 | MVK-F-Homo | TGGACCTCAGCTTACCCAACA | 21 |
| 14 | MVK-R-Homo | GACTGAAGCCTGGCCACATC | 20 |
| 15 | PMVK-F-Homo | GGCAAGAGGAAATCCGGGAA | 20 |
| 16 | PMVK-R-Homo | CCTCCTTGTAGGTGCTGGTG | 20 |
| 17 | MVD-F-Homo | TGAACTCCGCGTGCTCATC | 19 |
| 18 | MVD-R-Homo | CGGTACTGCCTGTCAGCTTCT | 21 |
| 19 | FDPS-F-Homo | CTTCCTATAGCTGCAGCCATGTAC | 24 |
| 20 | FDPS-R-Homo | GCATTGGCGTGCTCCTTCT | 19 |
| 21 | SQLE-F-Homo | CGTGCTCCTCTTGGTACCTCAT | 22 |
| 22 | SQLE-R-Homo | CGGTCAAGGCGGAGATTATC | 20 |
| 23 | FDFT1-F-Homo | TCAGACCAGTCGCAGTTTCG | 20 |
| 24 | FDFT1-R-Homo | CTGCGTTGCGCATTTCC | 17 |
| 25 | GGPS1-F-Homo | GGGAAGGATTCATGTAGGCATCG | 23 |
| 26 | GGPS1-R-Homo | TGGTTCTCACTTGTTTACCTGG | 22 |
