# Supplementary material for: MiR-122-5p regulates the mevalonate pathway by targeting p53 in non-small cell lung cancer
Source: Cell Death Dis. 2023 Apr 1;14(4):234. doi: 10.1038/s41419-023-05761-9 (PMC10067850; doi:10.1038/s41419-023-05761-9)
Supplement: Supplementary file 2 — Western blot raw data [file 41419_2023_5761_MOESM2_ESM.docx]

Full and uncropped western blot for Figure 2C

(β-actin; Lane (1) is on the figure)

(1)


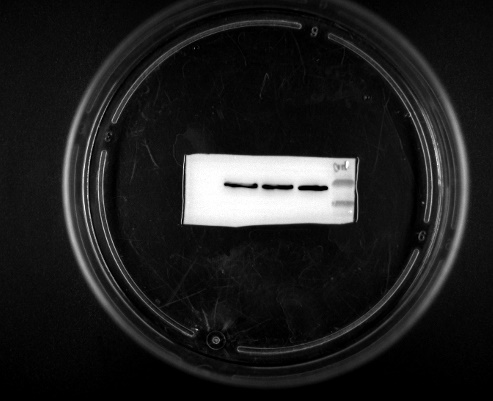


(2)


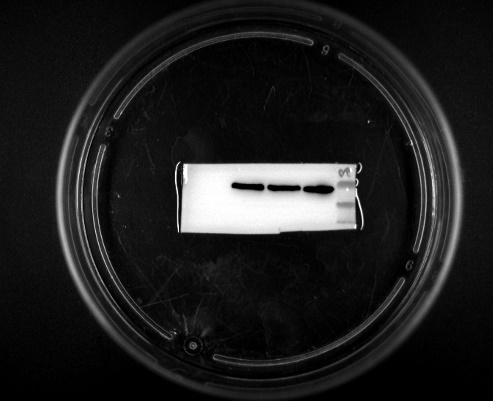


(3)


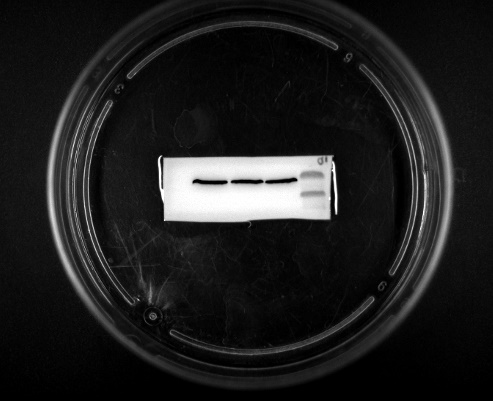


Full and uncropped western blot for Figure 2C

(p53; Lane (1) is on the figure)

(1)


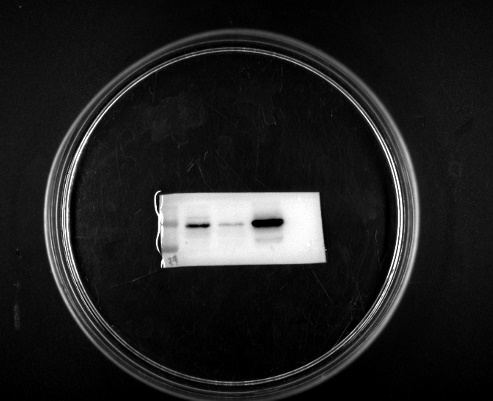


(2)


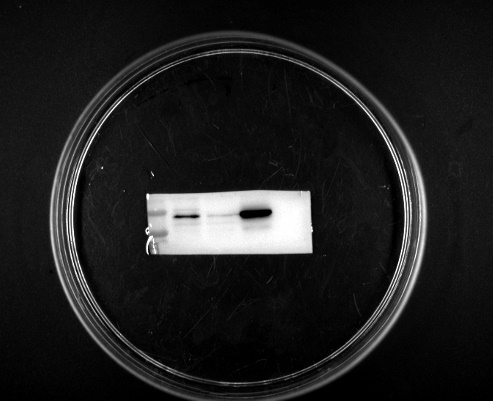


(3)


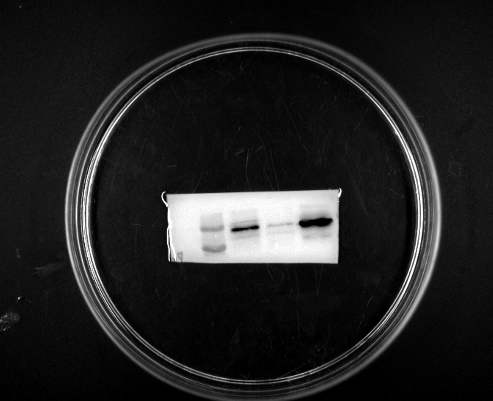


Full and uncropped western blot for Figure 2C

(ABCA1; Lane (1) is on the figure)

(1)


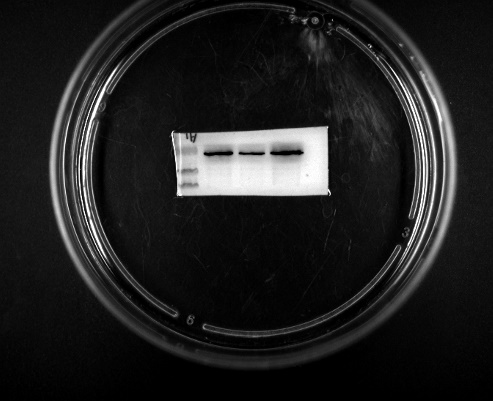


(2)


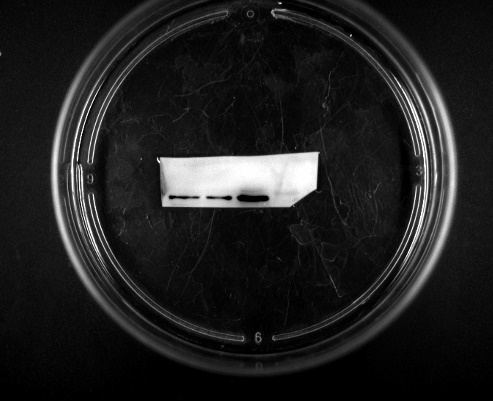


(3)


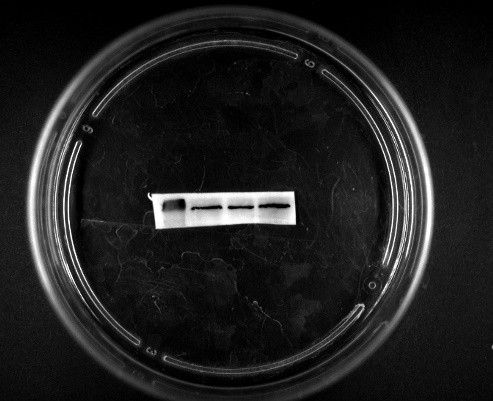


Full and uncropped western blot for Figure 2C

(SREBP2(M); Lane (1) is on the figure)

(1)


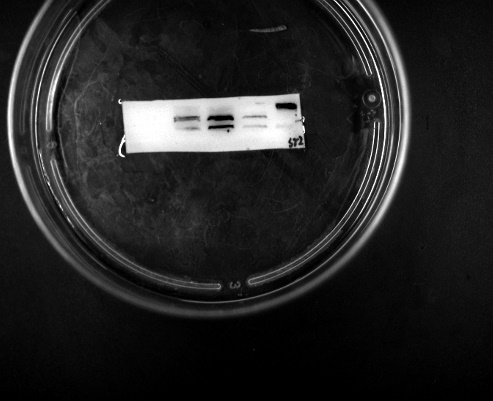


(2)


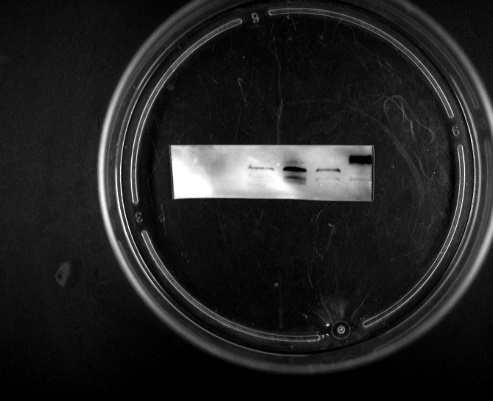


(3)


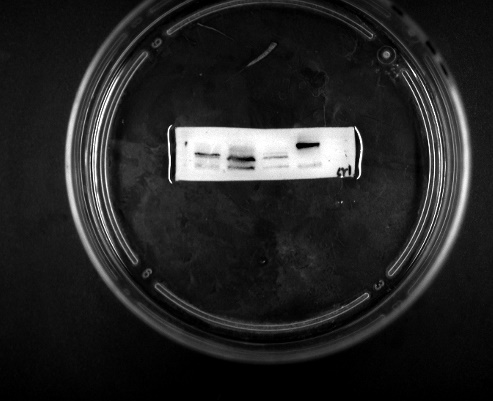


Full and uncropped western blot for Figure 2C

(SREBP2(P); Lane (1) is on the figure)

(1)
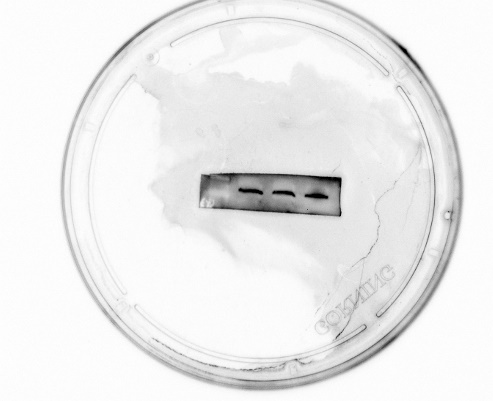

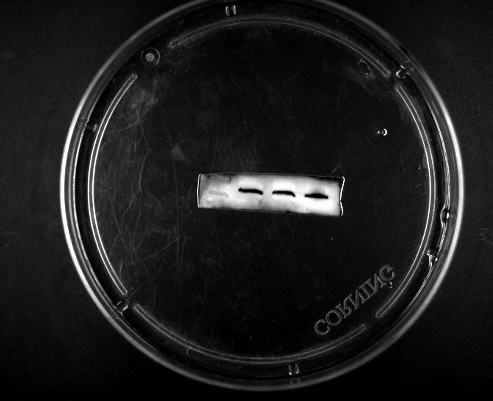


(2)


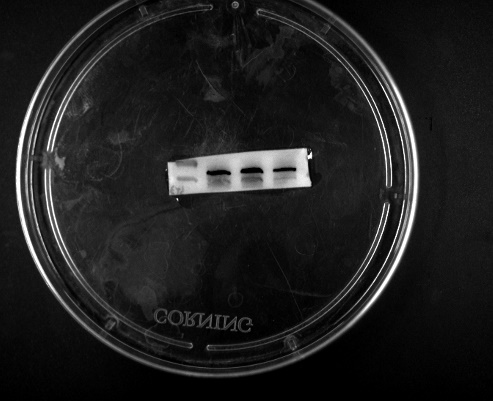


(3)


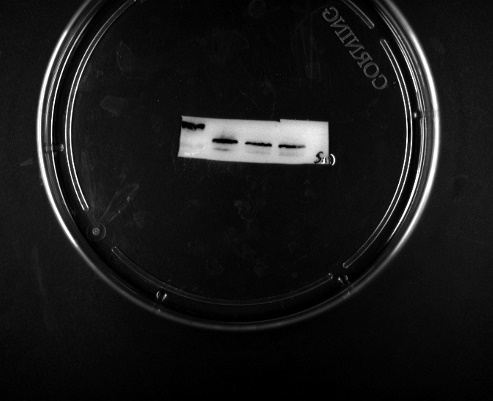


Full and uncropped western blot for Figure 2O

(β-actin; Lane (1) is on the figure)

(1)





(2)


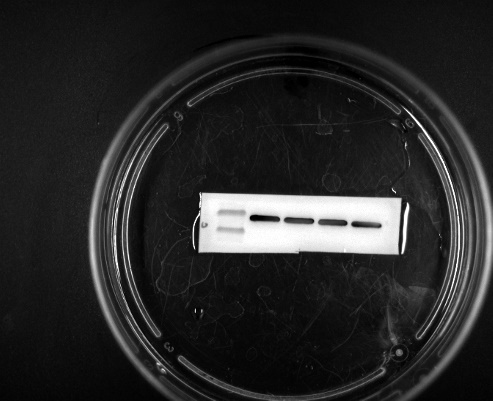


(3)


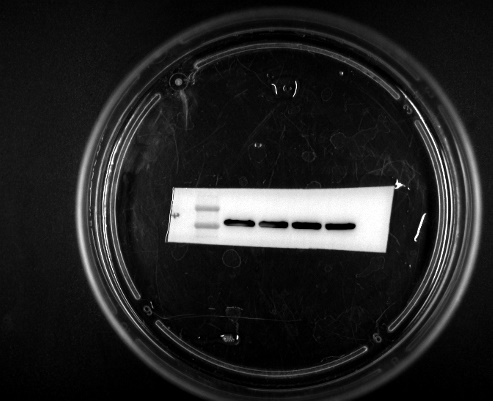


Full and uncropped western blot for Figure 2O

(FDFT1; Lane (1) is on the figure)

(1)


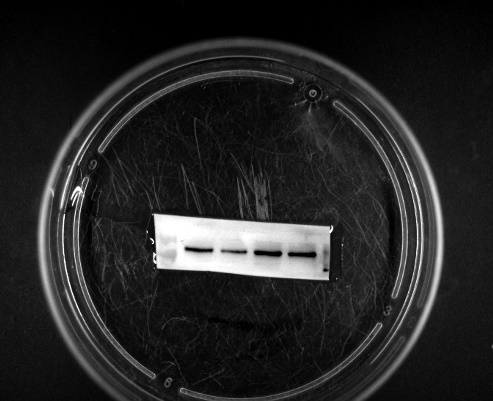


(2)


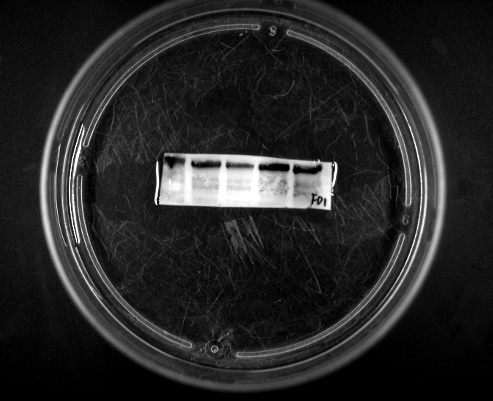


(3)


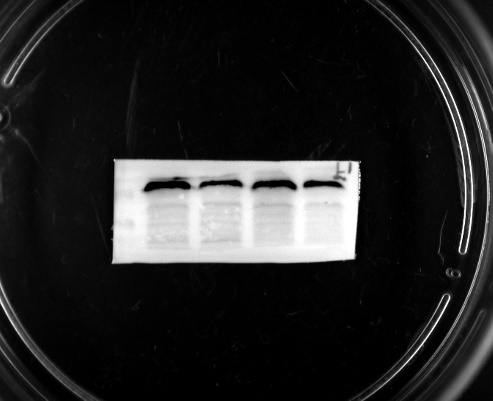


Full and uncropped western blot for Figure 2O

(HMGCR; Lane (1) is on the figure)

(1)


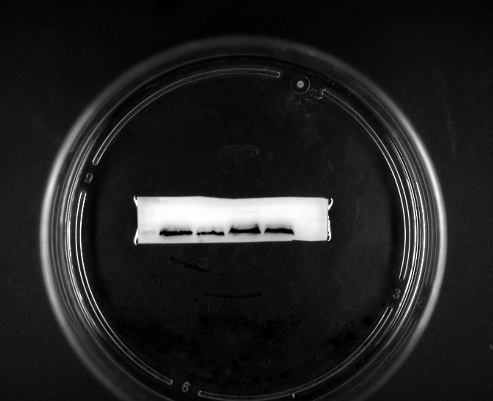


(2)


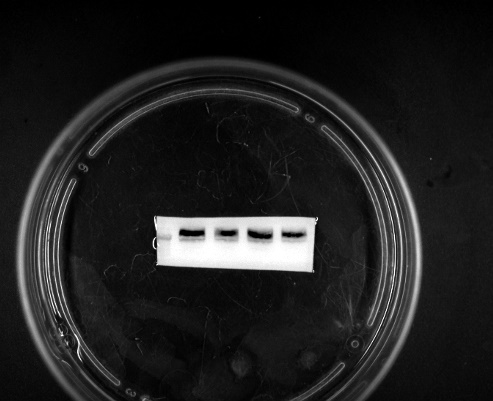


(3)


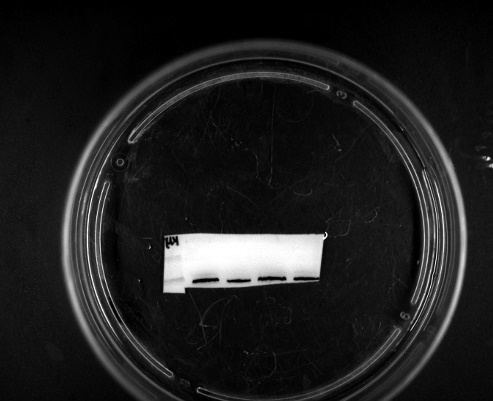


Full and uncropped western blot for Figure 2O

(HMGCS1; Lane (1) is on the figure)

(1)


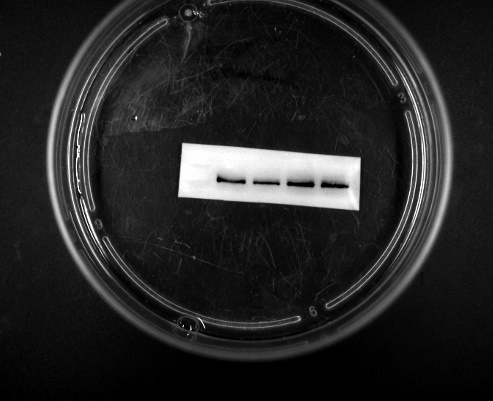


(2)


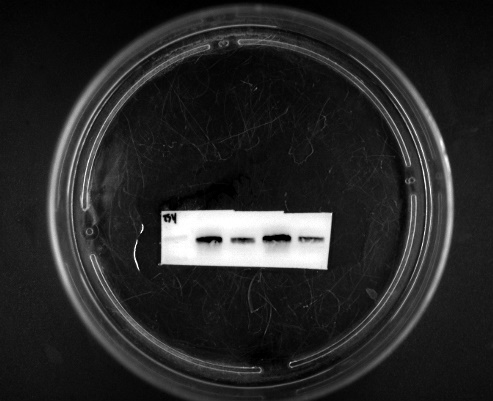


(3)


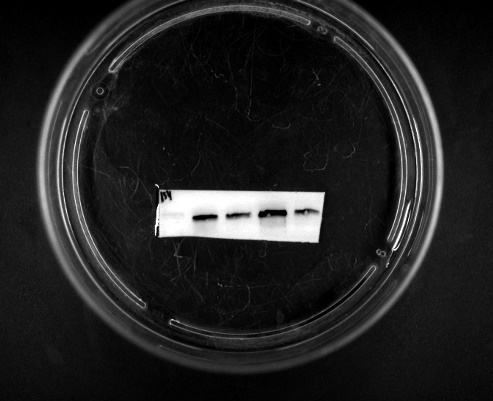


Full and uncropped western blot for Figure 3C

(β-actin; Lane (1) is on the figure)

(1)


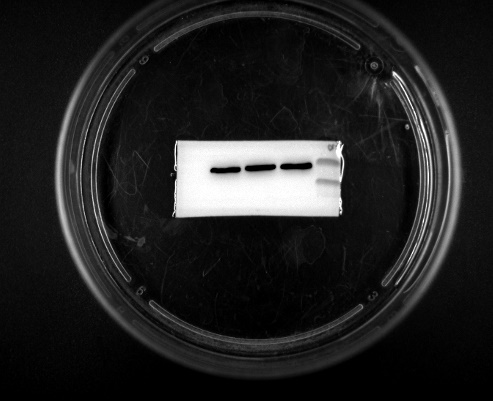


(2)


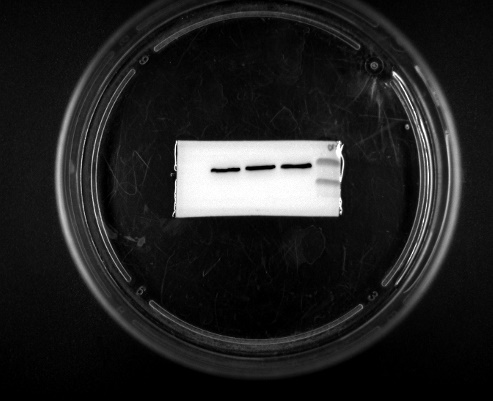


(3)


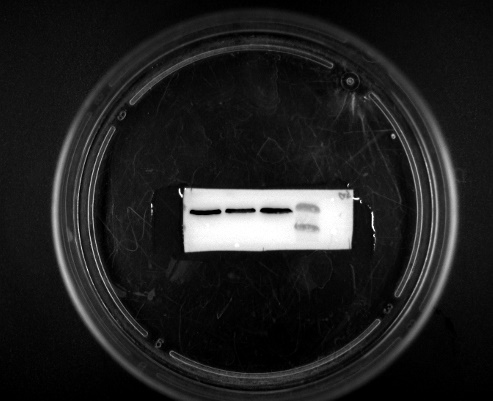


Full and uncropped western blot for Figure 3C

(p53; Lane (1) is on the figure)

(1)


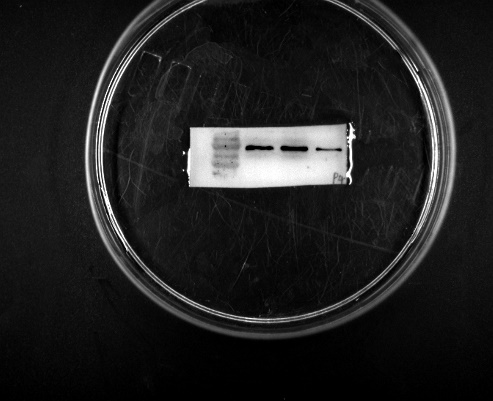


(2)


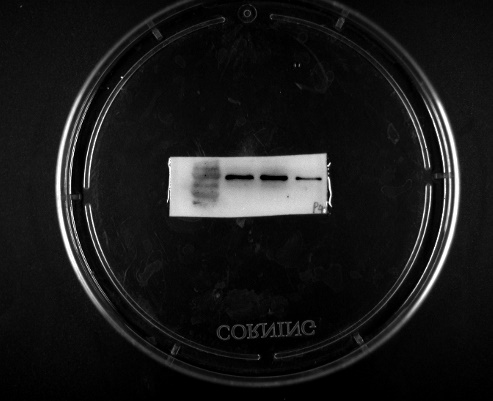


(3)


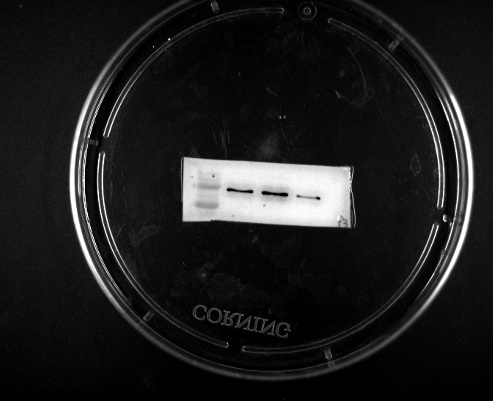


Full and uncropped western blot for Figure 3C

(ABCA1; Lane (1) is on the figure)

(1)


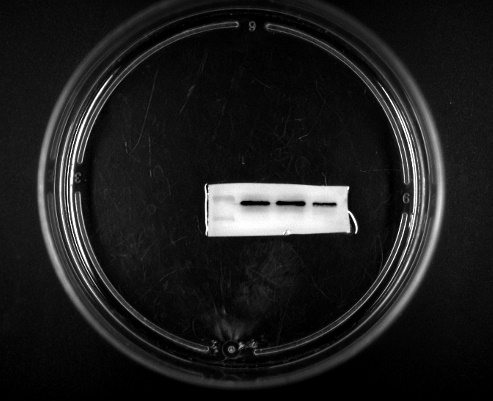


(2)


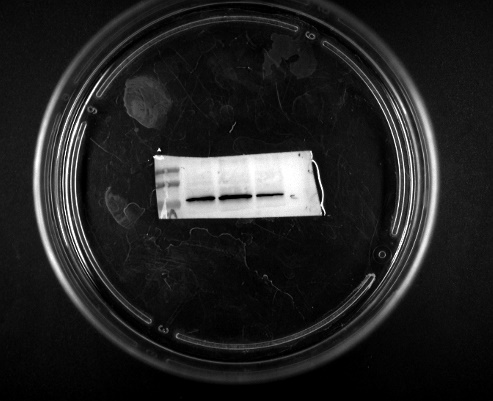


(3)


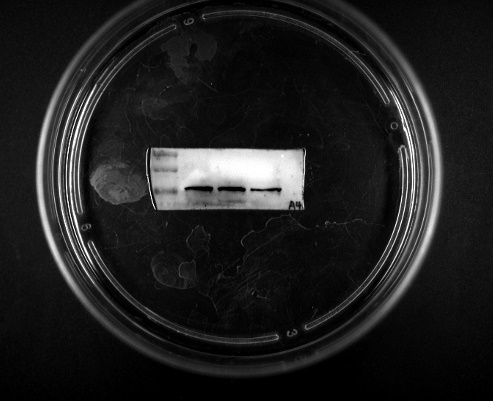


Full and uncropped western blot for Figure 3C

(SREBP2(M); Lane (1) is on the figure)

(1)


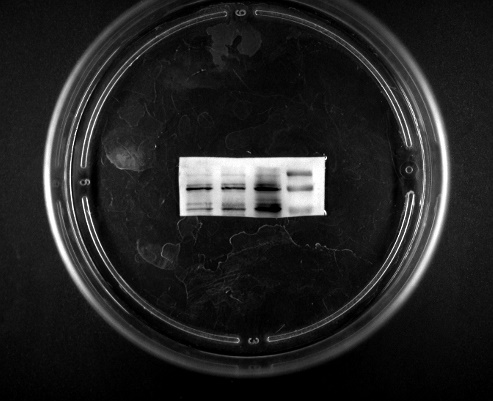


(2)


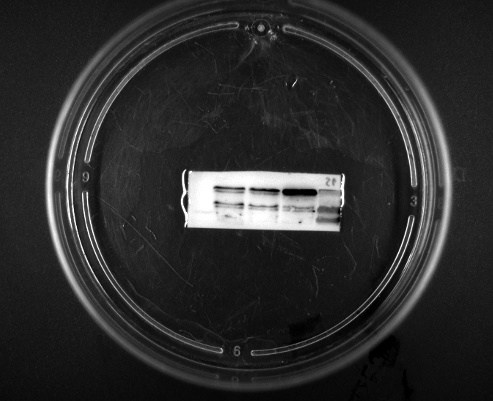


(3)


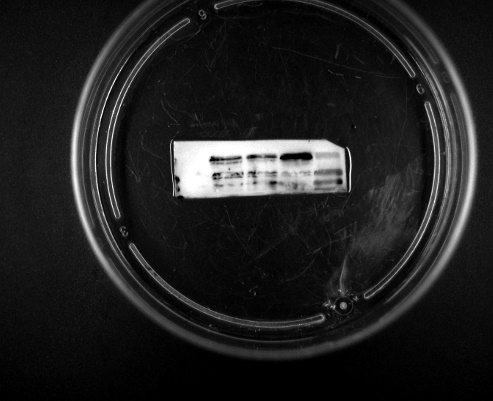


Full and uncropped western blot for Figure 3C

(SREBP2(P); Lane (1) is on the figure)

(1)


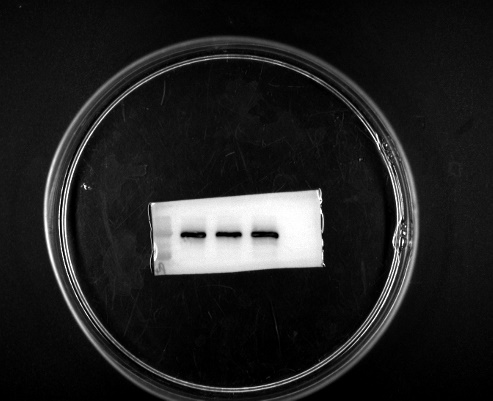


(2)


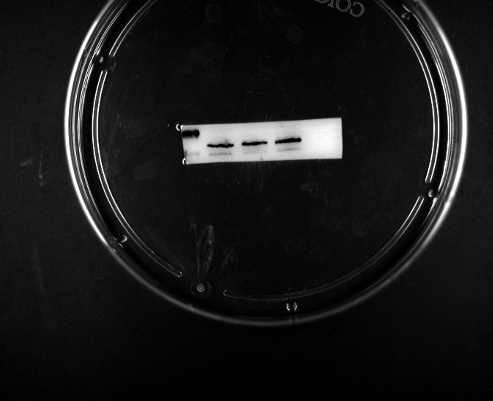


(3)


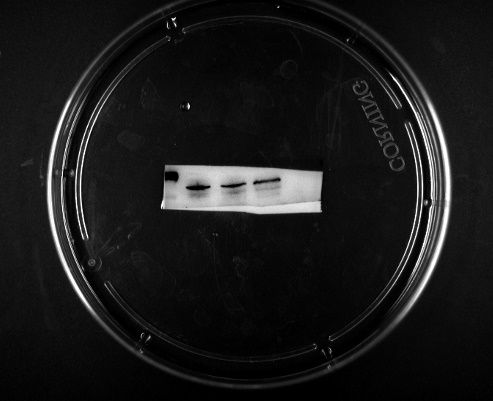


Full and uncropped western blot for Figure 3O

(β-actin; Lane (1) is on the figure)

(1)


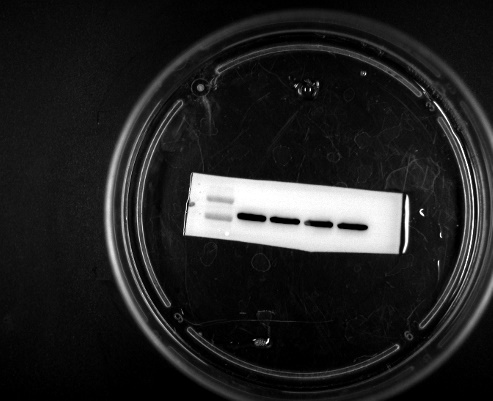


(2)


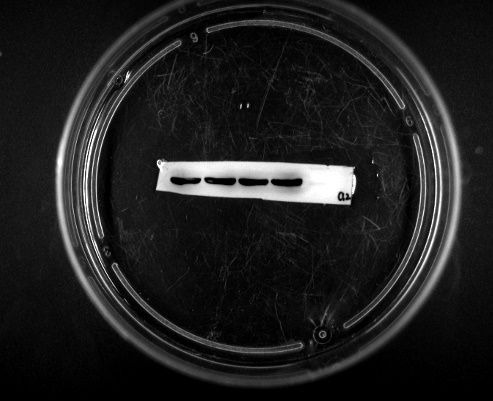


(3)


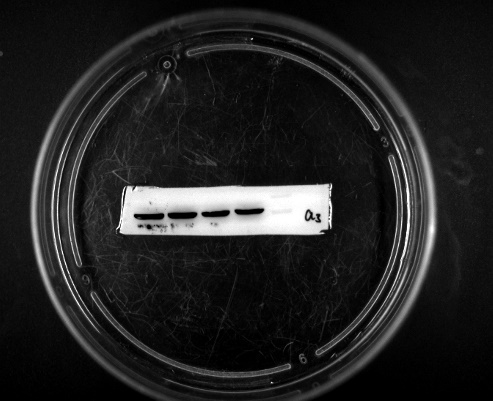


Full and uncropped western blot for Figure 3O

(FDFT1; Lane (1) is on the figure)

(1)


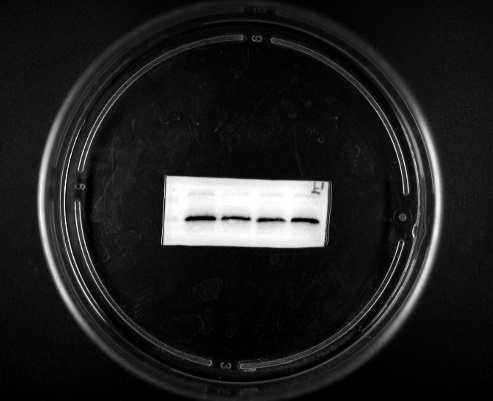


(2)


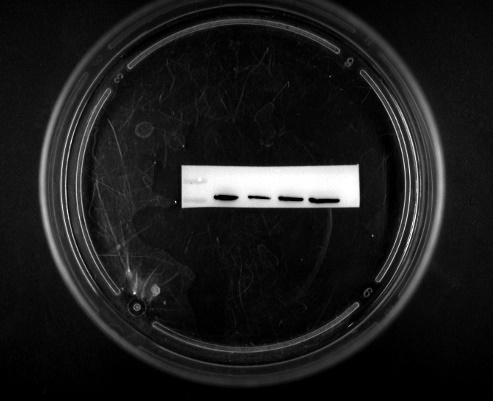


(3)


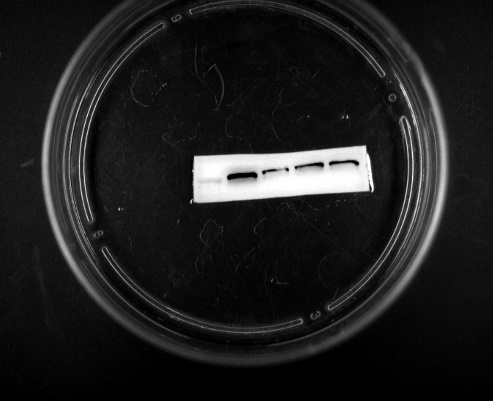


Full and uncropped western blot for Figure 3O

(HMGCR; Lane (1) is on the figure)

(1)


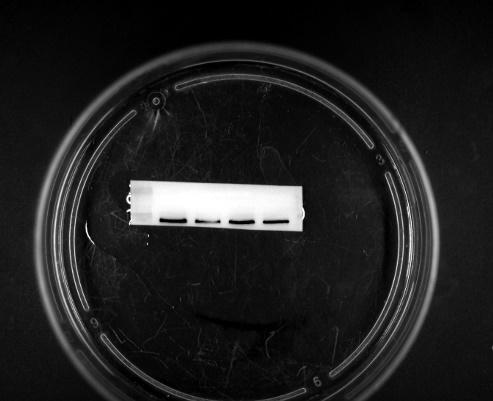


(2)


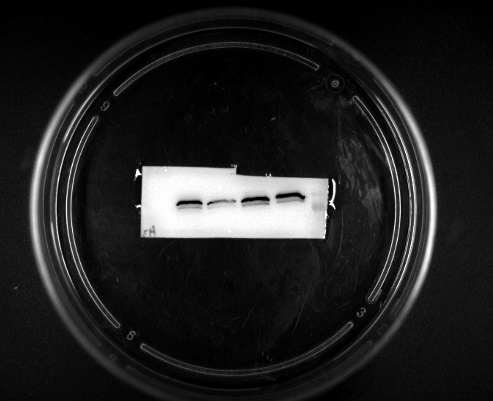


(3)

Full and uncropped western blot for Figure 3O

(HMGCS1; Lane (1) is on the figure)

(1)

(2)

(3)
